# Eriophyoid mites from Qinghai Province, northwestern China with descriptions of nine new species (Acari, Eriophyoidea)

**DOI:** 10.3897/zookeys.196.2726

**Published:** 2012-05-21

**Authors:** Hao-Sen Li, Xiao-Feng Xue, Xiao-Yue Hong

**Affiliations:** 1 Department of Entomology, Nanjing Agricultural University, Nanjing, Jiangsu 210095, China

**Keywords:** Eriophyoid mites, Qinghai, taxonomy, new species

## Abstract

Eriophyoid mites from Qinghai Province, northwestern China were studied herein. Up to now, only six species have been reported from Qinghai Province. In field surveys, 17 eriophyoid mite species were collected, among which nine species were found new to science. The new species and their host plants are listed as follows: *Acaphyllisa tuberculumae* **sp. n.** on *Populus* sp. (Salicaceae); *Proiectus xiningensis* **sp. n.** on *Pinus* sp. (Pinaceae); *Phyllocoptes beishaniensis* **sp. n.** on *Spiraea mongolica* Maxim. (Rosaceae); *Tetra pruniana* **sp. n.** on *Prunus tomentosa* Thunb. (Rosaceae) Rupr. (Berberidaceae); *Tetra pyriana* **sp. n.** on *Pyrus calleryana* Decne. (Rosaceae); *Tetra simonia* **sp. n.** on *Populus simonii* Carr. (Salicaceae); *Diptacus berberinus* **sp. n.** on *Berberis amurensis* Rupr. (Berberidaceae); *Diptacus mengdaensis* **sp. n.** on *Lonicera elisae* Franch. (Caprifoliaceae); *Rhyncaphytoptus spinus* **sp. n.** on *Lonicera rupicola* Hook. f. et Thoms. (Caprifoliaceae). *Aculops ulmi* Hong & Xue, 2005 was re-described.

## Introduction

Qinghai Province (89°35'E–103°04'E, 13°39'N–39°19'N), located in the northwest of the People’s Republic of China, is a part of the Qinghai-Tibet Plateau, with an average elevation of over 3000m (1650m–6860m). The average temperature ranges from 0.4°C to 7.4°C. (Qinghai Province Government Website).

Up to now, six species of eriophyoid mites from Qinghai have been reported. They are *Aceria paramacrodonis* Kuang, 1988 on *Lycium* sp. (Solanaceae), *Aceria qinghaiensis* Kuang, 1997 on *Salix babylonica* L. (Salicaceae), *Aculodes salicis* Kuang, 1997 on *Salix babylonica* L. (Salicaceae), *Aculops xiningensis* Kuang, 2000 on *Malus pumila* P. Mill. (Rosaceae), *Aculus huangzhongensis* Kuang, 2000 on *Syringa oblata* Lindl. (Oleaceae) and *Tetraspinus syringae* Lin & Kuang, 2001 on *Syringa oblata* Lindl. (Oleaceae). In July 2007, field surveys were conducted in Qinghai Province, northwestern China. Twenty-five eriophyoid mite samples were collected and 17 eriophyoid mite species were identified, among which nine species were found new to science. No species reported earlier from Qinghai were collected in this survey. In total, there are 23 species of the Eriophyoidea from Qinghai Province, belonging to two families and 12 genera. A list of eriophyoid mites from Qinghai Province is given (Table 1).

**Table 1.** List of eriophyoid mites and their hosts in Qinghai Province.

Family	Subfamily	Tribe	Species	Host
Eriophyidae	Eriophyinae	Aceriini	*Aceria paramacrodonis* Kuang, 1988	*Lycium* sp. (Solanaceae)
			*Aceria qinghaiensis* Kuang, 1997	*Salix babylonica* L. (Salicaceae)
	Phyllocoptinae	Acaricalini	*Acaphyllisa tuberculumae* sp. n.	*Populus* sp. (Salicaceae)
		Phyllocoptini	*Proiectus xiningensis* sp. n.	*Pinus* sp. (Pinaceae)
			*Phyllocoptes beishaniensis* sp. n.	*Spiraea mongolica* Maxim. (Rosaceae)
			*Phyllocoptes asperatae* Song, Xue & Hong, 2006	*Picea meyeri* Rehd. Et Wils. (Pinaceae)
			*Phyllocoptes dangchangi* Song, Xue & Hong, 2006	*Picea* sp. (Pinaceae)
			*Phyllocoptes gansunensis* Kuang & Luo, 1998	*Potentilla parvifolia* Fisch. ap. Lehm. (Rosaceae)
			*Phyllocoptruta platycladusa* Xue, Song, Amrine & Hong, 2007	*Juniperus chinensis* L. (Cupressaceae)
		Anthocoptini	*Aculus changbais* Xue, Song & Hong, 2008	*Salix chaenomeloides* Kimura ( Salicaceae)
			*Aculus huangzhongensis* Kuang, 2000	*Syringa oblata* Lindl. (Oleaceae)
			*Aculodes salicis* Kuang, 1997	*Salix babylonica* L. (Rosaceae)
			*Aculops umli* Hong & Xue, 2005	*Ulmus* sp. (Ulmaceae)
			*Aculops xiningensis* Kuang, 2000	*Malus pumila* P. Mill. (Rosaceae)
			*Tetraspinus syringae* Lin & Kuang, 2001	*Syringa oblata* Lindl. (Oleaceae)
			*Tetra pinnatifidae* Xue, Song & Hong, 2006	*Prunus armeniaca* Linn. (Rosaceae)
			*Tetra pruniana* sp. n.	*Prunus tomentosa* Thunb. (Rosaceae)
			*Tetra pyriana* sp. n.	*Pyrus calleryana* Decne. (Rosaceae)
				*Pyrus betulifolia* Bunge. (Rosaceae)
			*Tetra simonia* sp. n.	*Populus simonii* Carr. (Salicaceae)
Diptilomiopidae	Diptilomiopinae		*Diptacus berberinus* sp. n.	*Berberis amurensis* Rupr. (Berberidaceae)
			*Diptacus mengdaensis* sp. n.	*Lonicera elisae* Franch. (Caprifoliaceae)
	Rhyncaphytoptinae		*Rhyncaphytoptus spinus* sp. n.	*Lonicera rupicola* Hook. f. et Thoms. (Caprifoliaceae)
			*Rhyncaphytoptus ulmi* Xin & Dong, 1981	*Ulmus* sp. (Ulmaceae)

## Materials and methods

In the field, eriophyoid mites were collected by the aid of a hand-lens (30X) from the lower surface of host plant leaves. Eriophyoid mites, together with host plants, were immersed in 75% alcohol and kept in vials. Each vial was marked with the collection data, such as specimen number, collection date, host plant, mite color, location, collector, and mite relationship to host plant. The collection data were also recorded in the collection notebook for further use. The host plants were kept in a plant specimen folder in a dry environment.

The morphological terminology used here follows that of Lindquist (1996) and the generic classification was made according to Amrine et al. (2003). Slides were mounted using Keifer’s F-medium and modified Berlese medium (Amrine and Manson 1996). Specimens were measured based on the methods outlined by de Lillo et al. (2010). Specimens were examined with a Zeiss A2 (Germany) research microscope with phase contrast and semi-schematic drawings were made. Photos of slide mounted mites were taken with the same microscope (100× oil immersion objective with 10× eyepieces), connected to a computer using Axiovision image analysis software. For each species, the holotype female measurement precedes the corresponding range for paratypes (given in parentheses). All measurements are in micrometres (μm), and are lengths when not otherwise specified. All the type materials are deposited at Arthropod/Mite collection of, the Department of Entomology, Nanjing Agricultural University, Jiangsu Province, China.

## Taxonomy

### Family Eriophyidae Nalepa, 1898. Subfamily Eriophyinae Nalepa, 1898. Tribe Aceriini Amrine & Stasny, 1994. Genus *Aceria* Keifer, 1944

#### Aceria paramacrodonis

Kuang, 1988

http://species-id.net/wiki/Aceria_paramacrodonis

Aceria paramacrodonis Kuang 1988: 49–50, figures 1–6.
Aceria paramacrodonis ; Amrine and Stasny 1994: 73.
Aceria paramacrodonis ; Kuang 1995: 61, figure 45.
Aceria paramacrodonis ; Amrine and Stasny 1996: 295–304.
Aceria paramacrodonis ; Hong and Zhang 1996: 25, figure 43.
Aceria paramacrodonis ; Song et al. 2008: 4.

##### Host.

*Lycium* sp. (Solanaceae).

##### Relation to host.

Leaf gall; mites produce pocket galls on the lower side of leaves.

##### Distribution.

China (Gansu, Ningxia, Qinghai, Shandong).

#### Aceria qinghaiensis

Kuang, 1997

http://species-id.net/wiki/Aceria_qinghaiensis

Aceria qinghaiensis Kuang and Pang 1997: 231–232, figures 6–11.
Aceria qinghaiensis ; Kuang et al. 2005: 31–32, figure 29.
Aceria qinghaiensis ; Song et al. 2008: 14.

##### Host.

*Salix babylonica* L. (Salicaceae).

##### Relation to host. 

The mites produce pockets on the lower surface of the leaves.

##### Distribution.

China (Gansu, Qinghai).

### Subfamily Phyllocoptinae Nalepa, 1892. Tribe Acaricalini Amrine & Stasny, 1994. Genus *Acaphyllisa* Keifer, 1978

#### Acaphyllisa tuberculumae sp. n.

urn:lsid:zoobank.org:act:90E42ADF-5C3A-449D-A4C1-BA012B3D09F1

http://species-id.net/wiki/Acaphyllisa_tuberculumae

**Figure 1.**
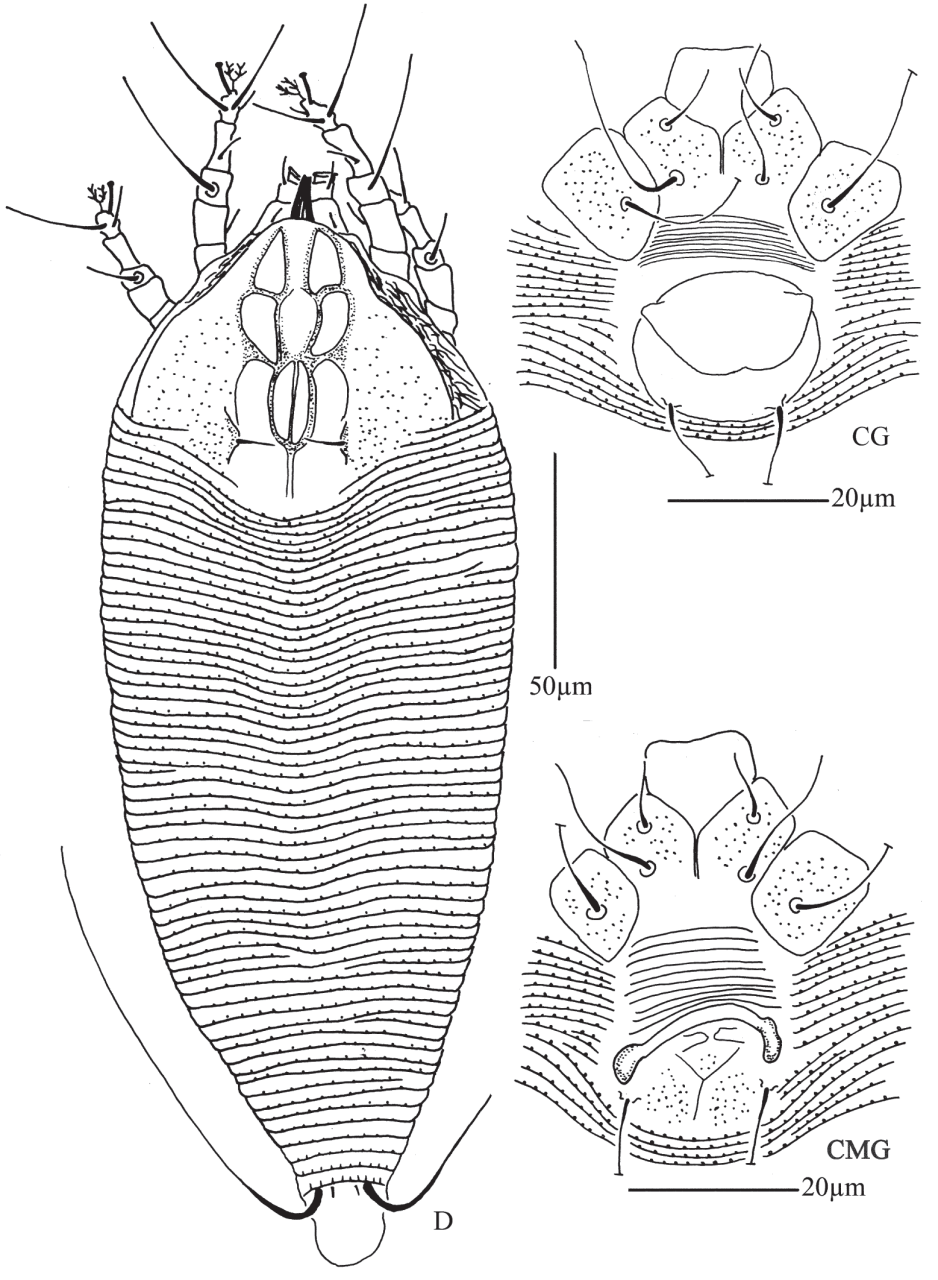
*Acaphyllisa tuberculumae* sp. n.: **D** dorsal view of female **CG** coxae and female genitalia **CMG** coxae and male genitalia.

Figures 1 [Fig F2] –3

##### Description.

Female. (n = 8) Body fusiform, light yellow, 171 (171–195), 72 (70–75) wide. **Gnathosoma** 21 (20–21), projecting obliquely down, suboral plate present, pedipalp coxal seta (*ep*) 4 (4–5), dorsal pedipalp genual seta (*d*) 7 (7–8), cheliceral stylets 16 (16–18). **Prodorsal shield** 49 (45–49), 53 (53–60) wide, subtriangular; frontal lobe 6 (5–8); median, admedian and submedian lines present, median line ending at basal 1/2 of prodorsal shield, admedian lines connected at basal 1/2 and 2/3 of prodorsal shield, forming three cells on each side of the median line. Scapular tubercles ahead of rear shield margin, 2 (2–3), 18 (18–19) apart, scapular setae (*sc*) 8 (6–8), projecting centrad. **Coxigenital region** with 10 smooth annuli. Coxisternal plates with granules, anterolateral setae on coxisternum **I** (*1b*) 7 (7–8), 14 (14–15) apart, proximal setae on coxisternum **I** (*1a*) 19 (19–21), 11 (10–11) apart, proximal setae on coxisternum **II** (*2a*) 51 (51–53), 26 (26–28) apart, tubercles *1b* and *1a* 8 (8–9) apart, tubercles *1a* and *2a* 8 (8–9) apart. Prosternal apodeme combined, 7 (6–7). **Leg I** 36 (35–36), femur 10 (9–10), basiventral femoral seta (*bv*) 8 (8–10); genu 5 (5–6), antaxial genual seta (*l"*) 29 (29–31); tibia 8 (7–8), paraxial tibial seta (*l’*) 7 (7–8), located at 1/4 from dorsal base; tarsus 8 (7–8), seta *ft’* 17 (17–19), seta *ft"* 22 (22–23), seta *u’* 5 (5–6); tarsal empodium (*em*) 6 (6–7), divided, 2-rayed on each side, tarsal solenidion (*ω*) 7 (6–7), knobbed. **Leg II** 28 (28–33), femur 10 (9–10), basiventral femoral seta (*bv*) 10 (9–10); genu 5 (4–5), antaxial genual seta (*l"*) 6 (6–8); tibia 6 (6–7); tarsus 7 (6–7), seta *ft’* 5 (5–7), seta *ft"* 21 (21–22), seta *u’* 5 (5–6); tarsal empodium (*em*) 6 (5–6), divided, 2-rayed on each side, tarsal solenidion (*ω*) 6 (6–7), knobbed. **Opisthosoma** dorsally with 55 (55–57) annuli, with round microtubercles, ventrally with 77 (74–77) annuli, with round microtubercles. Setae *c2* 31 (28–31) on ventral annulus 14 (14–16), 56 (54–56) apart; setae *d* 55 (55–60) on ventral annulus 34 (33–34), 38 (33–38) apart; setae *e* 17 (16–17) on ventral annulus 52 (52–53), 20 (18–20) apart; setae *f* 28 (25–28) on ventral annulus 71 (69–71), 26 (25–26) apart. Setae *h1* 2 (2–3), *h2* 70 (70–75). **Female genitalia** 18 (17–18), 24 (24–25) wide, coverflap smooth, setae *3a* 33 (33–35), 14 (14–15) apart.

Male. (n = 1) Body fusiform, light yellow, 150, 57 wide. **Gnathosoma** 17, projecting obliquely down, suboral plate present, pedipalp coxal seta (*ep*) 5, dorsal pedipalp genual seta (*d*) 5, cheliceral stylets 13. **Prodorsal shield** has the same design as female, 44, 48 wide, subtriangular; frontal lobe 5. Scapular tubercles ahead of rear shield margin, 3, 18 apart, scapular setae (*sc*) 6, projecting centrad. **Coxigenital region** with 10 smooth annuli. Coxisternal plates with granules, anterolateral setae on coxisternum **I** (*1b*) 7, 12 apart, proximal setae on coxisternum **I** (*1a*) 18, 10 apart, proximal setae on coxisternum **II** (*2a*) 45, 23 apart, tubercles *1b* and *1a* 7 apart, tubercles *1a* and *2a* 7 apart. Prosternal apodeme combined, 5. **Legs** with usual series of setae. **Leg I** 29, femur 9, basiventral femoral seta (*bv*) 8; genu 5, antaxial genual seta (*l"*) 31; tibia 7, paraxial tibial seta (*l’*) 6, located at 1/4 from dorsal base; tarsus 6, seta *ft’* 17), seta *ft"* 18, seta *u’* 4; tarsal empodium (*em*) 6, divided, 2-rayed on each side, tarsal solenidion (*ω*) 6, knobbed. **Leg II** 28, femur 10, basiventral femoral seta (*bv*) 8; genu 4, antaxial genual seta (*l"*) 8; tibia 6; tarsus 6, seta *ft’* 5, seta *ft"* 18, seta *u’* 4; tarsal empodium (*em*) 5, divided, 2-rayed on each side, tarsal solenidion (*ω*) 6, knobbed. **Opisthosoma** dorsally with 51 annuli, with round microtubercles, ventrally with 66 annuli, with round microtubercles. Setae *c2* 21 on ventral annulus 12, 42 apart; setae *d* 42 on ventral annulus 27, 28 apart; setae *e* 16 on ventral annulus 43, 16 apart; setae *f* 25 on ventral annulus 61, 21 apart. Setae *h1* 2, *h2* 60. **Male genitalia** forming a “Y” like structure in the middle, 19 wide, setae *3a* 22, 15 apart.

**Figure 2. F2:**
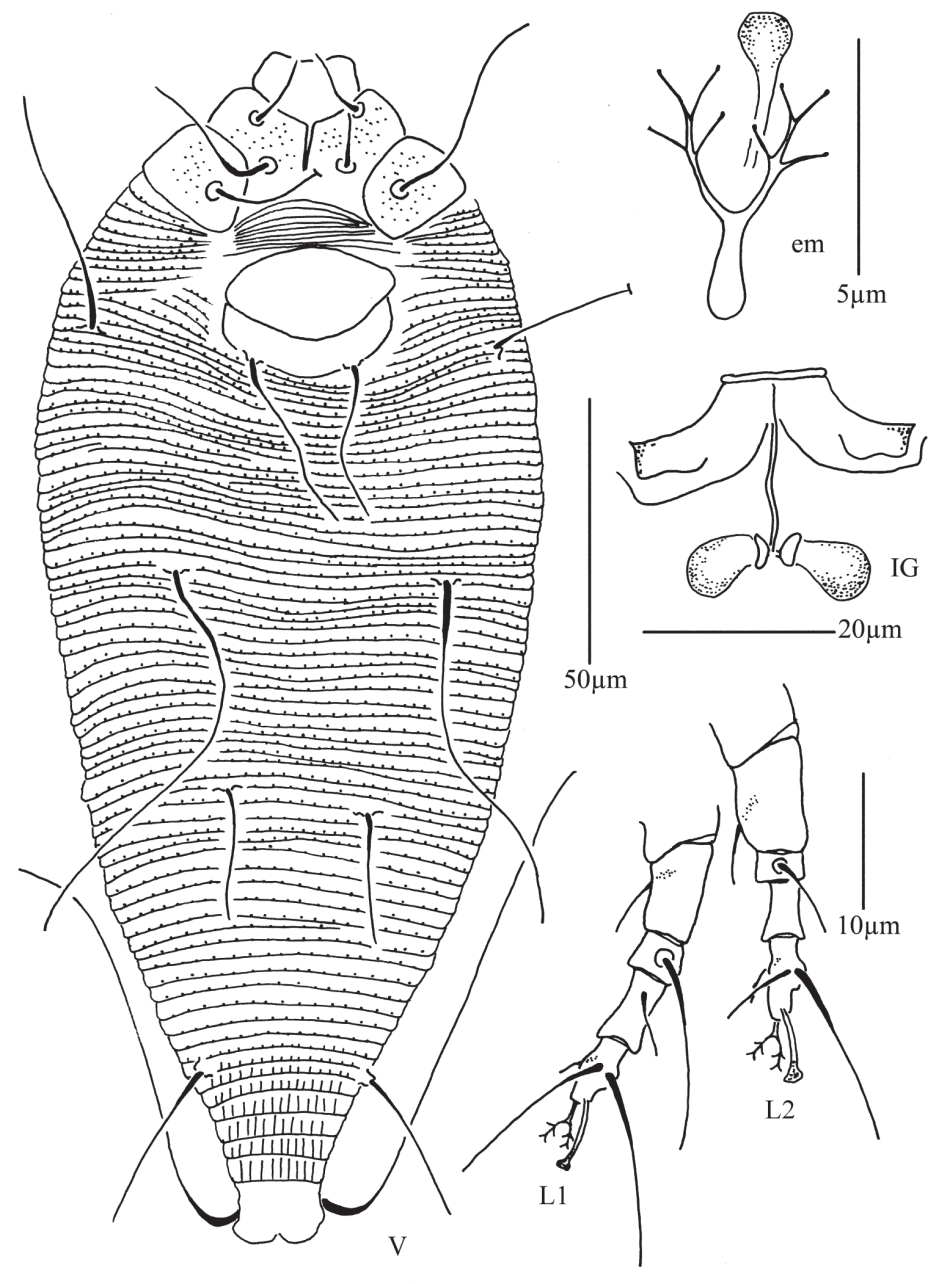
*Acaphyllisa tuberculumae* sp. n.: **V** ventral view of female **em** empodium **IG** female internal genitalia **L1** leg I **L2** leg II.

**Figure 3.**
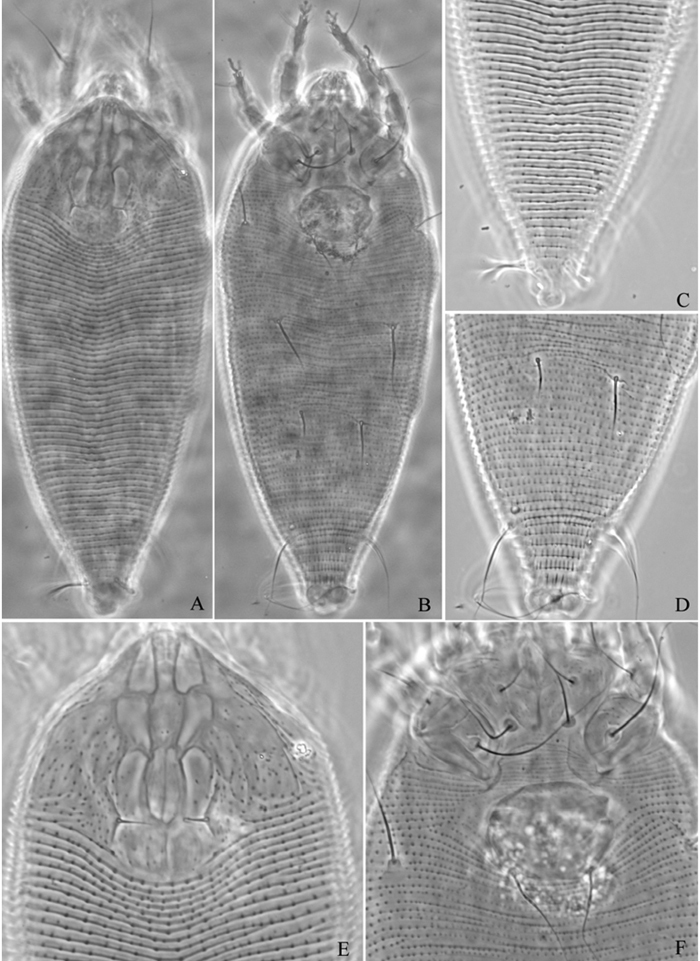
*Acaphyllisa tuberculumae* sp. n.: **A** dorsal view of female **B** ventral view of female **C** dorsal view of female posterior part **D** ventral view of female posterior part **E** prodorsal shield **F** coxae and female genitalia.

##### Type material.

**Holotype**, female (slide number NJAUEri789B, marked Holotype), from *Populus* sp. (Salicaceae), Xining City, Qinghai Province, P. R. China, 36°38'18"N, 101°45'27"E, elevation 2241m, 21 July 2007, coll. Xiao-Feng Xue. **Paratypes**, 7 females and 1 male (slide number NJAUEri789B), with the same data as holotype.

##### Relation to host.

Vagrant on leaf lower surface. No damage to the host was observed.

##### Etymology.

The specific designation *tuberculumae* is from the character of dorsal opisthosomal microtubercles, “tuberculum” in Latin; masculine in gender.

##### Differential diagnosis.

This species is similar to *Acaphyllisa populi* Xue & Hong, 2006, but can be differentiated from the latter by prodorsal shield with six cells in the middle (prodorsal shield without cells in *Acaphyllisa populi*), opisthosoma dorsally with round microtubercles (opisthosoma dorsally with elliptical microtubercles only on ridges in *Acaphyllisa populi*), female genitalia coverflap smooth (female genital coverflap with 10 longitudinal ridges in *Acaphyllisa populi*).

### Tribe Phyllocoptini Nalepa, 1892. Genus *Proiectus* Huang, 2001

#### Proiectus xiningensis sp. n.

urn:lsid:zoobank.org:act:21DA68FE-18B9-4305-B088-20BF3A865A3C

http://species-id.net/wiki/Proiectus_xiningensis

**Figure 4.**
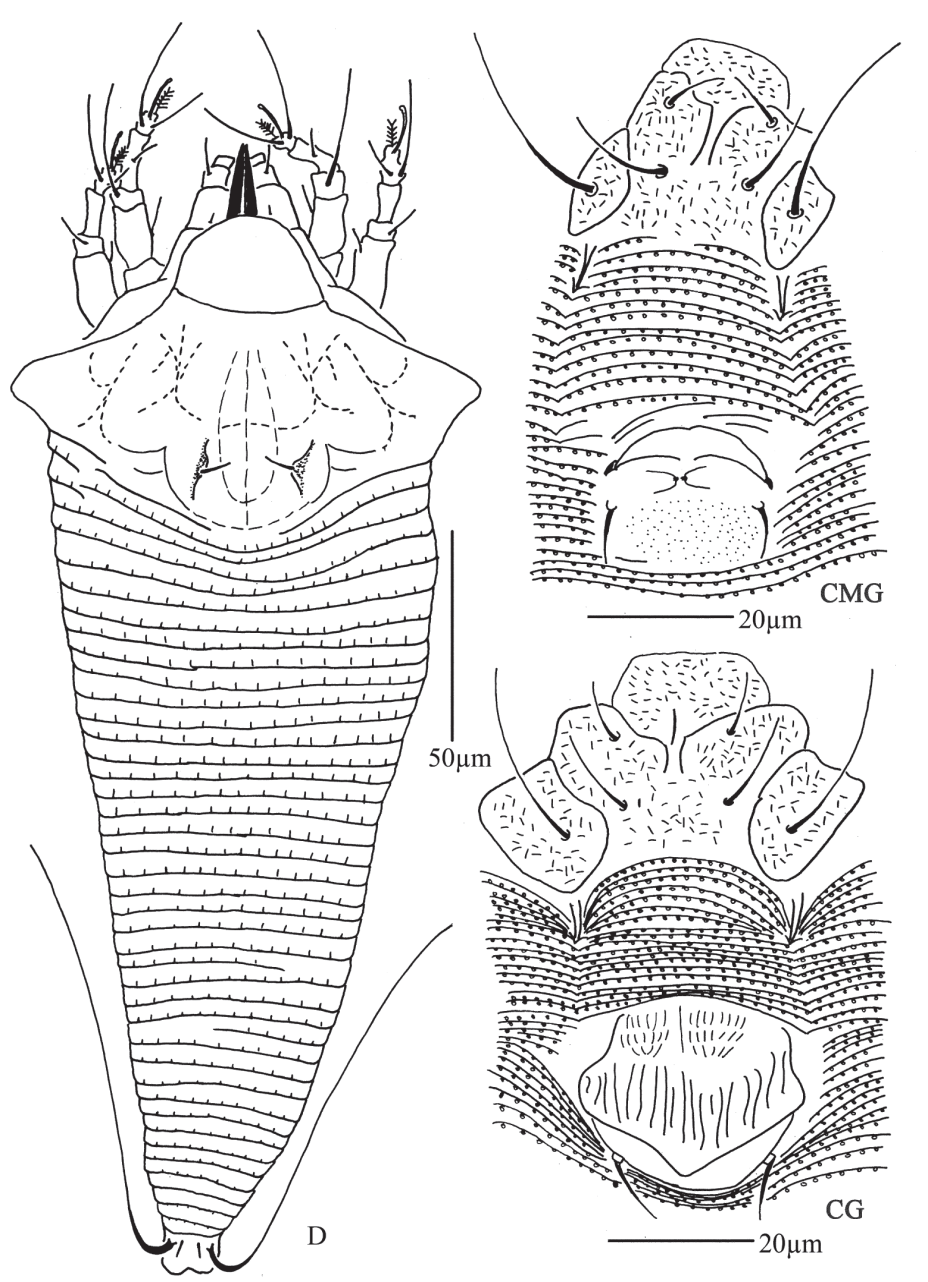
*Proiectus xiningensis* sp. n.: **D** dorsal view of female **CMG** coxae and male genitalia **CG** coxae and female genitalia.

Figures 4 [Fig F5] [Fig F6] –7

##### Description.

Female. (n = 5) Body fusiform, light yellow, 248 (223–308), 100 (100–110) wide, 90 (90–91) thick. **Gnathosoma** 33 (33–34), projecting obliquely down, suboral plate present, pedipalp coxal seta (*ep*) 3 (3–5), dorsal pedipalp genual seta (*d*) 15 (11–15), cheliceral stylets 33 (33–34). **Prodorsal shield** 73 (65–73), 100 (100–110) wide, subtriangular, with a large projection on each lateral margin 7 (6–7); frontal lobe broad 24 (22–24); median, admedian and submedian lines obscure, median and admedian lines connected at base. Scapular tubercles ahead of rear shield margin, 2 (2–3), 30 (27–30) apart, scapular setae (*sc*) 8 (6–8), projecting centrad. **Coxigenital region** with 17 (14–17) annuli, with round microtubercles, with deep seam under coxisternal plate II. Coxisternal plates with short lines, anterolateral setae on coxisternum **I** (*1b*) 10 (10–11), 18 (18–19) apart, proximal setae on coxisternum **I** (*1a*) 22 (20–22), 13 (12–13) apart, proximal setae on coxisternum **II** (*2a*) 52 (50–52), 32 (32–33) apart, tubercles *1b* and *1a* 11 (10–11) apart, tubercles *1a* and *2a* 10 (10–11) apart. Prosternal apodeme separated, 4 (4–5). **Leg I** 43 (40–45), femur 9 (8–10), basiventral femoral seta (*bv*) 14 (12–14); genu 9 (8–10), antaxial genual seta (*l"*) 28 (28–34); tibia 15 (13–15), paraxial tibial seta (*l’*) 5 (5–6), located at 1/2 from dorsal base; tarsus 10 (9–10), seta *ft’* 17 (17–18), seta *ft"* 30 (30–32), seta *u’* 5 (5–6); tarsal empodium (*em*) 8 (7–8), simple, 5-rayed, tarsal solenidion (*ω*) 10 (9–10), knobbed. **Leg II** 40 (40–41), femur 13 (13–16), basiventral femoral seta (*bv*) 13 (10–13); genu 6 (6–8), antaxial genual seta (*l"*) 8 (7–8); tibia 10 (10–12); tarsus 8 (8–9), seta *ft’* 9 (8–9), seta *ft"* 27 (27–28), seta *u’* 5 (5–6); tarsal empodium (*em*) 8 (7–8), simple, 5-rayed, tarsal solenidion (*ω*) 10 (9–10), knobbed. **Opisthosoma** dorsally with 41 (39–41) annuli, with weak filamentous microtubercles, ventrally with 92 (92–100) annuli, with round microtubercles. Setae *c2* 21 (21–22) on ventral annulus 15 (15–20), 70 (70–73) apart; setae *d* 100 (80–100) on ventral annulus 34 (34–39), 37 (37–46) apart; setae *e* 55 (55–65) on ventral annulus 56 (56–61), 21 (21–22) apart; setae *f* 33 (30–33) on ventral annulus 84 (84–90), 28 (28–29) apart. Setae *h1* 7 (6–7), *h2* 65 (55–65). **Female genitalia** 28 (28–30), 33 (33–34) wide, coverflap with 19 longitudinal ridges, setae *3a* 10 (10–12), 23 (21–22) apart.

Male. (n = 1) Body fusiform, light yellow, 250, 90 wide. **Gnathosoma** 34, projecting obliquely down, suboral plate present, pedipalp coxal seta (*ep*) 3, dorsal pedipalp genual seta (*d*) 11, cheliceral stylets 50. **Prodorsal shield** has the same design as female, 70, 90 wide, subtriangular; with a large projection on each lateral margin, 6; frontal lobe broad, 21. Scapular tubercles ahead of rear shield margin, 2, 23 apart, scapular setae (*sc*) 6, projecting centrad. **Coxigenital region** with 16 annuli, with round microtubercles, with deep seam under coxisternal plate II. Coxisternal plates with short lines, anterolateral setae on coxisternum **I** (*1b*) 10, 15 apart, proximal setae on coxisternum **I** (*1a*) 15, 12 apart, proximal setae on coxisternum **II** (*2a*) 52, 29 apart, tubercles *1b* and *1a* 10 apart, tubercles *1a* and *2a* 11 apart. Prosternal apodeme separated, 5. **Leg I** 40, femur 10, basiventral femoral seta (*bv*) 13; genu 8, antaxial genual seta (*l"*) 31; tibia 13, paraxial tibial seta (*l’*) 5, located at 1/2 from dorsal base; tarsus 8, seta *ft’* 17, seta *ft"* 30, seta *u’* 5; tarsal empodium (*em*) 7, simple, 5-rayed, tarsal solenidion (*ω*) 9, knobbed. **Leg II** 37, femur 15, basiventral femoral seta (*bv*) 12; genu 6, antaxial genual seta (*l"*) 7; tibia 10; tarsus 8), seta *ft’* 7, seta *ft"* 25, seta *u’* 5; tarsal empodium (*em*) 7, simple, 5-rayed, tarsal solenidion (*ω*) 9, knobbed. **Opisthosoma** dorsally with 41 annuli, with weak filamentous microtubercles, ventrally with 94 annuli, with round microtubercles. Setae *c2* 21 on ventral annulus 18, 60 apart; setae *d* 80 on ventral annulus 36, 38 apart; setae *e* 60 on ventral annulus 58, 20 apart; setae *f* 27 on ventral annulus 88, 25 apart. Setae *h1* 6, *h2* 65. **Male genitalia** 25 wide, setae *3a* 12, 22 apart.

**Figure 5. F5:**
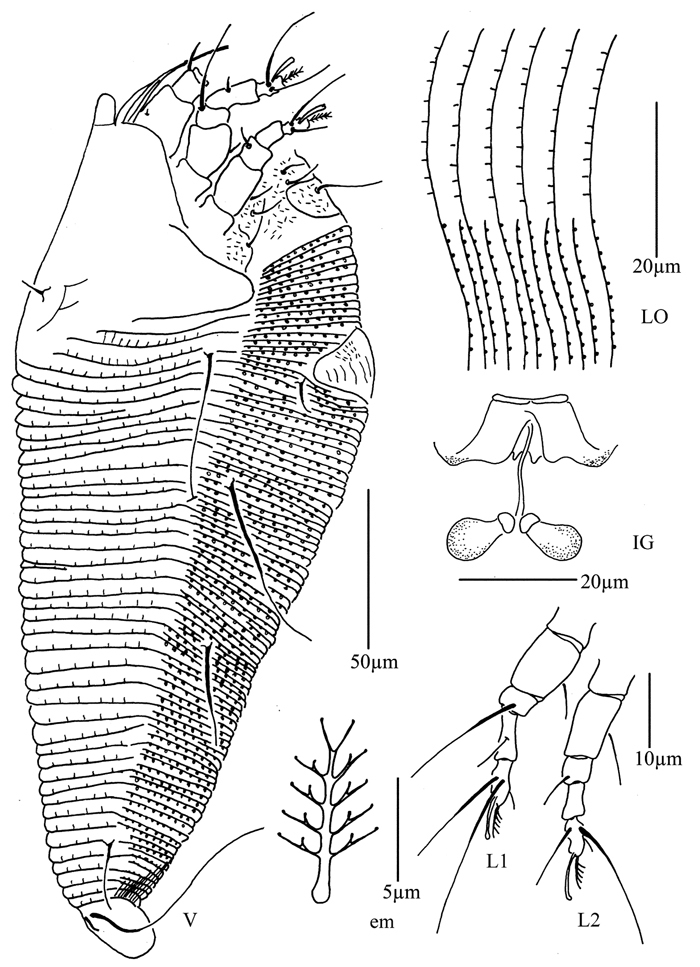
*Proiectus xiningensis* sp. n.: **L** lateral view of female **LO** lateral microtubercles **IG** female internal genitalia **em** empodium **L1** leg I **L2** leg II.

**Figure 6. F6:**
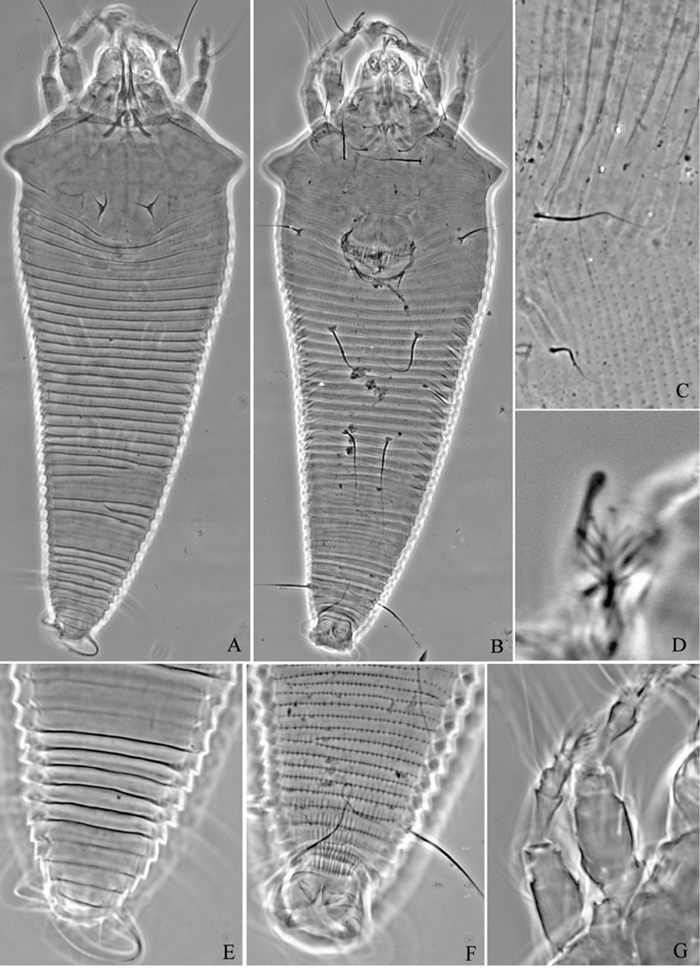
*Proiectus xiningensis* sp. n.: **A** dorsal view of female **B** ventral view of female **C** lateral microtubercles **D** empodium **E** dorsal view of female posterior part **F** ventral view of female posterior part **G **leg I and leg II.

**Figure 7.**
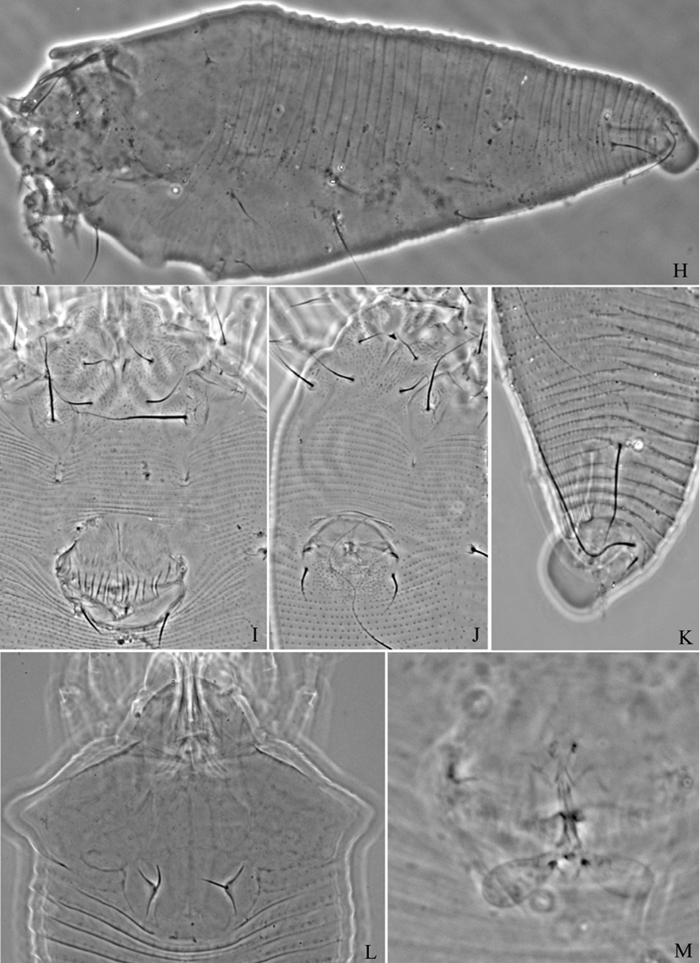
*Proiectus xiningensis* sp. n.: **H** lateral view of female **I** coxae and female genitalia **J** coxae and male genitalia **K** lateral view of female posterior part **L** prodorsal shield **M** female internal genitalia.

##### Type material.

**Holotype**, female (slide number NJAUEri790, marked Holotype), from *Pinus* sp. (Pinaceae), Xining City, Qinghai Province, P. R. China, 36°38'18"N, 101°45'27"E, elevation 2241m, 21 July 2007, coll. Xiao-Feng Xue. **Paratypes**, 4 females and 1 male (slide number NJAUEri790), with the same data as holotype.

##### Relation to host.

Vagrant on terminal part of the needles. No damage to the host was observed.

**Etymology.** The specific designation *xiningensis* is from the place name Xining City, where this new species was collected; feminine in gender.

##### Differential diagnosis.

This species is similar to *Proiectus thunbergis* Xue, Song, Amrine & Hong, 2007, but can be differentiated from the latter by median and admedian lines of prodorsal shield simple (median and admedian lines with granules in *Proiectus thunbergis*), opisthosoma dorsally annuli with weak filamentous microtubercles (opisthosoma dorsally annuli with round microtubercles in *Proiectus thunbergis*), tarsal empodium (*em*) 5-rayed (4-rayed in *Proiectus thunbergis*).

### Genus *Phyllocoptes* Nalepa, 1887

#### Phyllocoptes beishaniensis sp. n.

urn:lsid:zoobank.org:act:0ABE4028-A29D-4B4A-B7FE-133603A01C99

http://species-id.net/wiki/Phyllocoptes_beishaniensis

**Figure 8.**
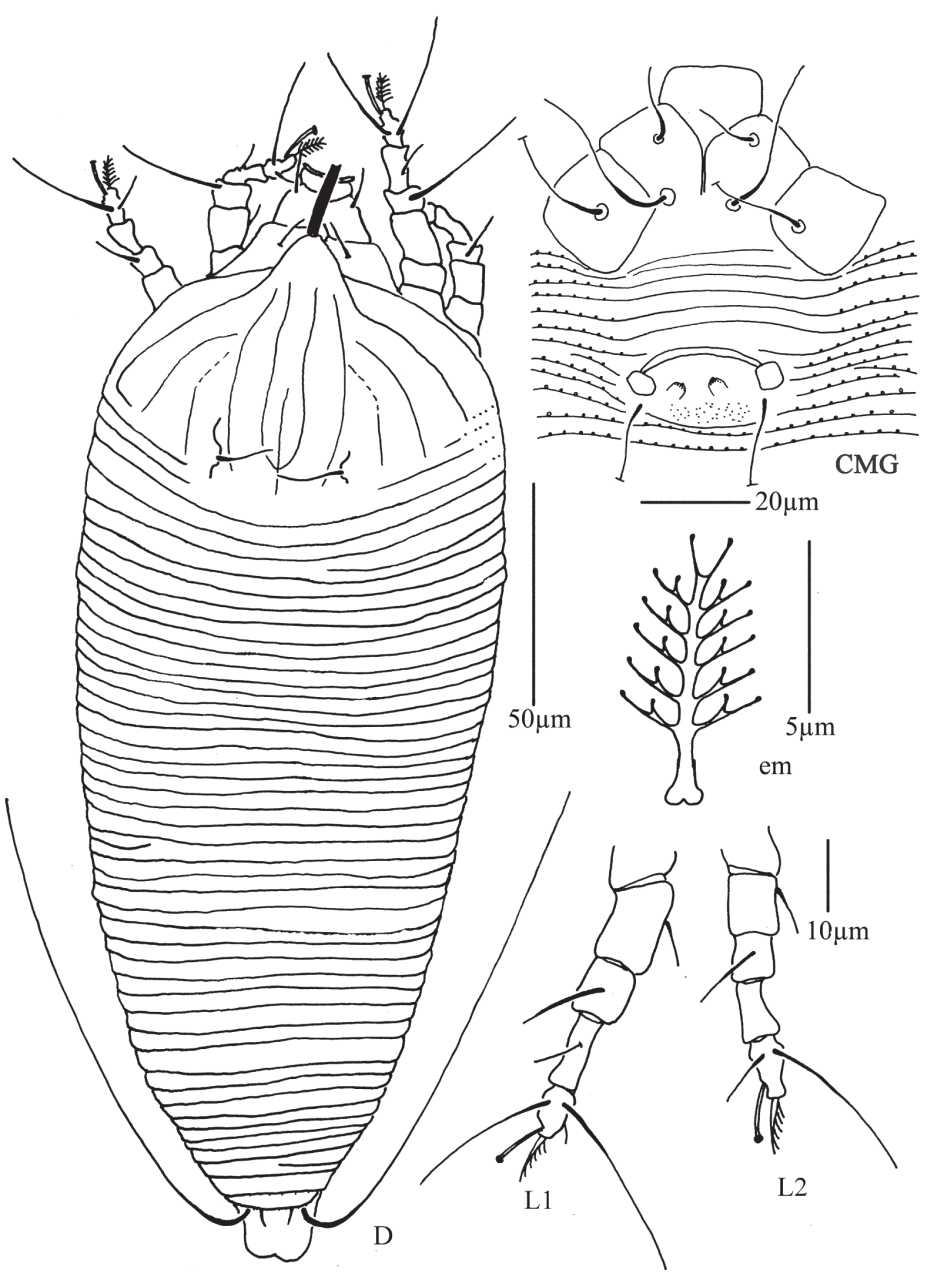
*Phyllocoptes beishaniensis* sp. n.: **D** dorsal view of female **CMG** coxae and male genitalia **em** empodium **L1** leg I **L2** leg II.

Figures 8 [Fig F9] [Fig F10] –11

##### Description.

Female. (n = 13) Body fusiform, light yellow, 168 (160–178), 63 (66–67) wide, 61 (61–68) thick. **Gnathosoma** 20 (20–25), projecting obliquely down, suboral plate present, pedipalp coxal seta (*ep*) 6 (5–6), dorsal pedipalp genual seta (*d*) 6 (6–7), cheliceral stylets 16 (16–20). **Prodorsal shield** 48 (46–48), 65 (60–65) wide, subtriangular; frontal lobe 10 (9–10); median, admedian and submedian lines present, median line ending at basal 1/2 of prodorsal shield and connected with admedian lines at basal 1/4. Scapular tubercles ahead of rear shield margin, 2 (2–3), 22 (21–22) apart, scapular setae (*sc*) 10 (8–11), projecting centrad. **Coxigenital region** with 5 (4–5) smooth annuli. Coxisternal plates smooth, anterolateral setae on coxisternum **I** (*1b*) 8 (7–8), 13 (13–14) apart, proximal setae on coxisternum **I** (*1a*) 21 (17–21), 10 (10–11) apart, proximal setae on coxisternum **II** (*2a*) 42 (42–45), 30 (30–31) apart, tubercles *1b* and *1a* 9 (9–10) apart, tubercles *1a* and *2a* 10 (10–11) apart. Prosternal apodeme combined 6 (6–7). **Leg I** 33 (33–34), femur 11 (10–11), basiventral femoral seta (*bv*) 12 (12–13); genu 6 (5–6), antaxial genual seta (*l"*) 23 (22–23); tibia 8 (8–9), paraxial tibial seta (*l’*) 7 (6–7), located at 1/3 from dorsal base; tarsus 6 (6–7), seta *ft’* 18 (18–19), seta *ft"* 22 (22–23), seta *u’* 5 (5–6); tarsal empodium (*em*) 8 (8–9), simple, 6-rayed, tarsal solenidion (*ω*) 7 (6–7), knobbed. **Leg II** 31 (29–31), femur 10 (10–11), basiventral femoral seta (*bv*) 10 (9–10); genu 6 (5–6), antaxial genual seta (*l"*) 10 (8–10); tibia 6 (6–7); tarsus 6 (6–7), seta *ft’* 7 (6–7), seta *ft"* 21 (21–22), seta *u’* 5 (5–6); tarsal empodium (*em*) 8 (8–9), simple, 6-rayed, tarsal solenidion (*ω*) 8 (8–9), knobbed. **Opisthosoma** dorsally with 45 (45–53) annuli, smooth; ventrally with 52 (51–52) annuli, with round microtubercles. Setae *c2* 30 (29–30) on ventral annulus 10 (9–13), 55 (54–55) apart; setae *d* 60 (55–60) on ventral annulus 20 (19–20), 30 (30–31) apart; setae *e* 40 (40–45) on ventral annulus 31 (30–31), 15 (15–16) apart; setae *f* 25 (25–27) on ventral annulus 46 (45–46), 24 (24–25) apart. Setae *h1* 5 (4–5), *h2* 80 (80–85). **Female genitalia** 17 (17–18), 22 (22–23) wide, coverflap with 10 longitudinal ridges, setae *3a* 56 (55–56), 16 (16–17) apart.

Male. (n = 9) Body fusiform, light yellow, 169–195, 56–67 wide. **Gnathosoma** 19–22, projecting obliquely down, suboral plate present, pedipalp coxal seta (*ep*) 4–5, dorsal pedipalp genual seta (*d*) 6–7, cheliceral stylets 17–18. **Prodorsal shield** has the same design as female, 42–50, 49–56 wide, subtriangular; frontal lobe 8–9. Scapular tubercles ahead of rear shield margin, 2–3, 18–19 apart, scapular setae (*sc*) 8–9, projecting centrad. **Coxigenital region** with 5 smooth annuli. Coxisternal plates smooth, anterolateral setae on coxisternum **I** (*1b*) 5–6, 12–15 apart, proximal setae on coxisternum **I** (*1a*) 13–14, 10–11 apart, proximal setae on coxisternum **II** (*2a*) 22–27, 27–31 apart, tubercles *1b* and *1a* 8–9 apart, tubercles *1a* and *2a* 9–10 apart. Prosternal apodeme combined, 5–7. **Leg I** 27–32, femur 10–11, basiventral femoral seta (*bv*) 11–12; genu 5–6, antaxial genual seta (*l"*) 21–22; tibia 6–7, paraxial tibial seta (*l’*) 5–6, located at 1/3 from dorsal base; tarsus 6–7, seta *ft’* 18–19, seta *ft"* 21–22, seta *u’* 5–6; tarsal empodium (*em*) 7–9, simple, 6-rayed, tarsal solenidion (*ω*) 6–7, knobbed. **Leg II** 27–30, femur 9–10, basiventral femoral seta (*bv*) 9–10; genu 5–6, antaxial genual seta (*l"*) 7–9; tibia 5–6; tarsus 5–6, seta *ft’* 5–6, seta *ft"* 17–19, seta *u’* 5–6; tarsal empodium (*em*) 7–8, simple, 6-rayed, tarsal solenidion (*ω*) 7–8, knobbed. **Opisthosoma** dorsally with 52 annuli, smooth, ventrally with 58 annuli, with round microtubercles. Setae *c2* 21–23 on ventral annulus 11, 46–53 apart; setae *d* 29–30 on ventral annulus 21, 25–28 apart; setae *e* 25–28 on ventral annulus 35, 12–13 apart; setae *f* 20 (20–23) on ventral annulus 54, 20–21 apart. Setae *h1* 4–5, *h2* 42–45. **Male genitalia** 19–21 wide, setae *3a* 13–14, 17–19 apart.

**Figure 9. F9:**
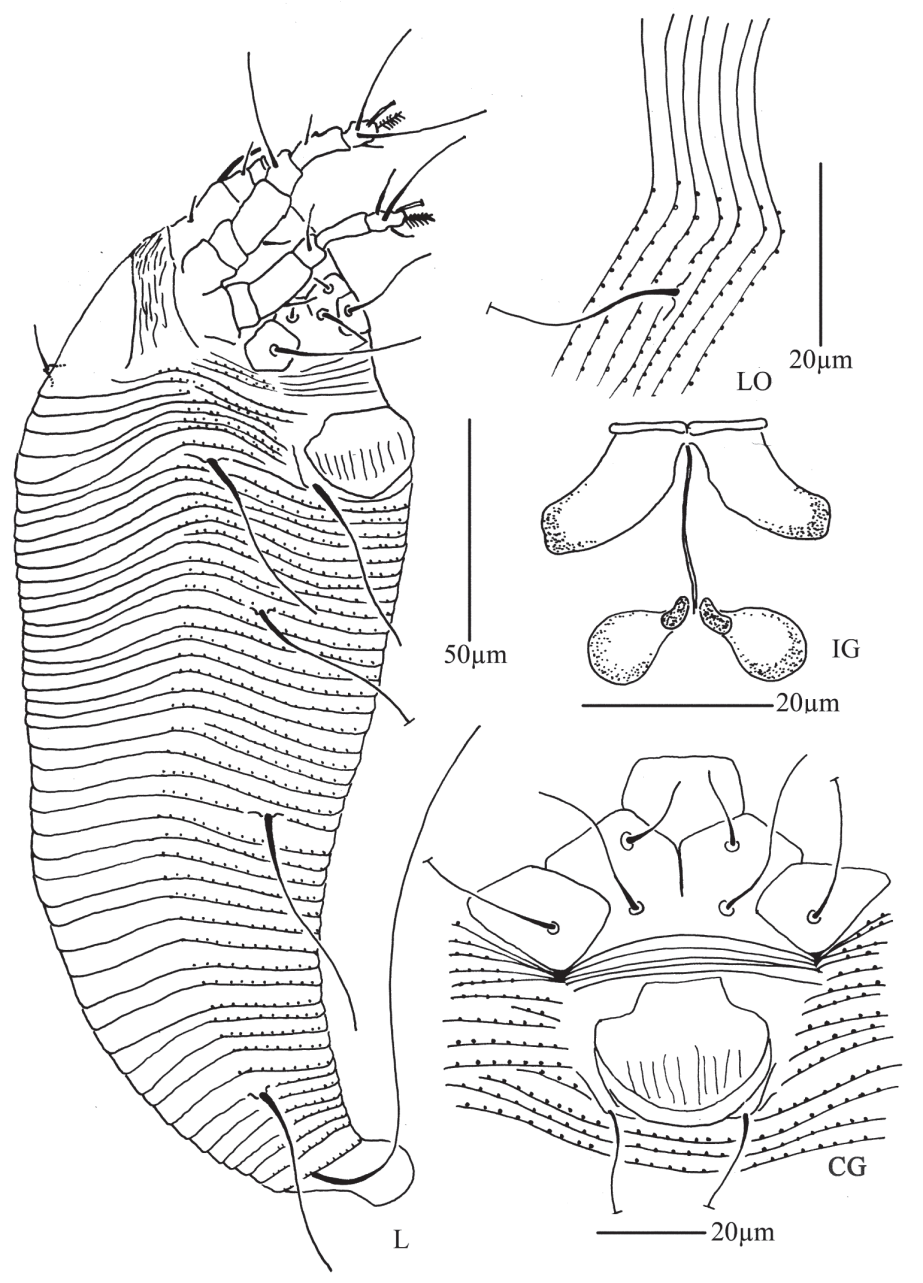
*Phyllocoptes beishaniensis* sp. n.: **L** lateral view of female **LO** lateral microtubercles **IG** female internal genitalia **CG** coxae and female genitalia

**Figure 10. F10:**
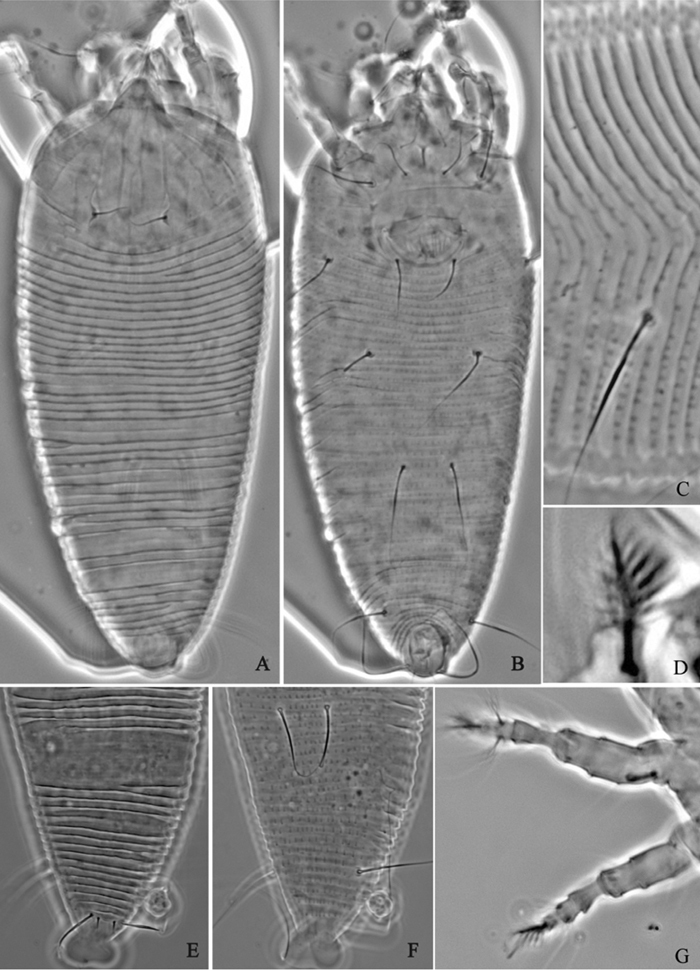
*Phyllocoptes beishaniensis* sp. n.: **A** dorsal view of female **B** ventral view of female **C** lateral microtubercles **D** empodium **E** dorsal view of female posterior part **F** ventral view of female posterior part **G** leg I and leg II.

**Figure 11.**
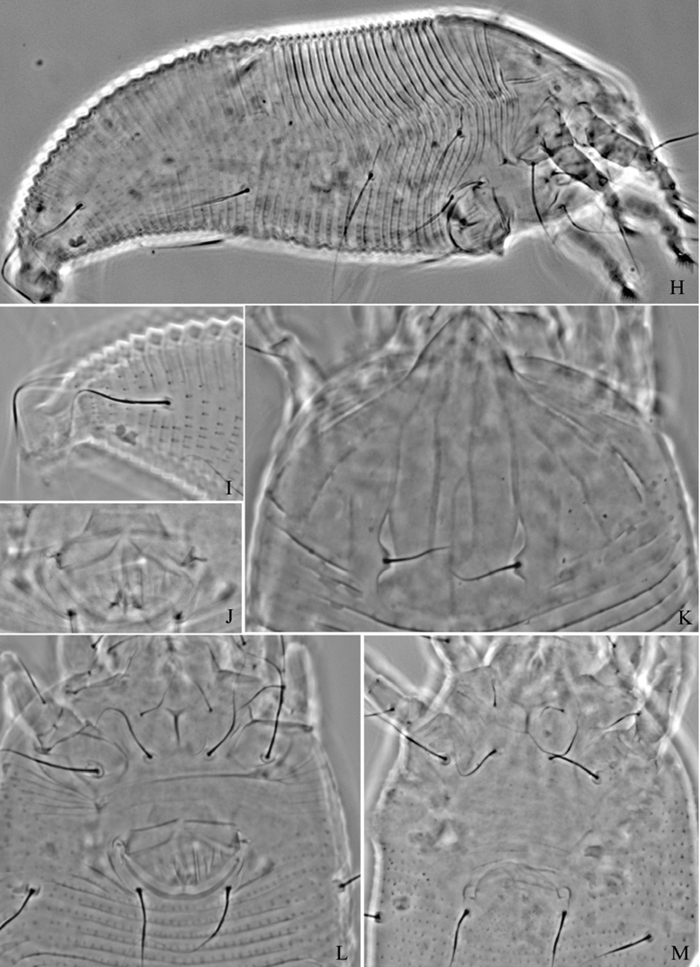
*Phyllocoptes beishaniensis* sp. n.: **H** lateral view of female **I** lateral view of female posterior part **J** female internal genitalia **K** prodorsal shield **L** coxae and female genitalia **M** coxae and male genitalia.

##### Type material.

**Holotype**, female (slide number NJAUEri815, marked Holotype), from *Spiraea mongolica* Maxim. (Rosaceae), Beishan National Forest Park, Huzhu County, Qinghai Province, P. R. China, 36°53'35"N, 102°25'56"E, elevation 2610m, 22 July 2007, coll. Xiao-Feng Xue. **Paratypes**, 7 females and 2 males (slide number NJAUEri815), with the same data as holotype.

##### Relation to host.

Vagrant on leaf lower surface.

##### Etymology.

The specific designation *beishaniensis* is from the place name Beishan National Forest Park, where the new species were collected; feminine in gender.

##### Differential diagnosis.

This species is similar to *Phyllocoptes adalius* (Keifer, 1939) from *Rosa* sp., but can be differentiated from the latter by prodorsal shield front lobe stout (prodorsal shield front lobe pointed in *Phyllocoptes adalius*), dorsal opisthosoma annuli smooth (opisthosoma annuli entirely covered with spinuliferous microtubercles in *Phyllocoptes adalius*), Coxisternal plates smooth (coxisternal plates with short lines in *Phyllocoptes adalius*).

#### Phyllocoptes asperatae

Song, Xue & Hong, 2006

http://species-id.net/wiki/Phyllocoptes_asperatae

Phyllocoptes asperatae Song et al. 2006: 36–38, figure 2.
Phyllocoptes asperatae ; Xue et al. 2009:134.
Phyllocoptes asperatae ; Song et al. 2009c: 37.

##### Material examined.

4 females and 2 males (slide number NJAUEri810), from a new host, *Picea meyeri* Rehd. Et Wils. (Pinaceae), Beishan National Forest Park, Huzhu County, Qinghai Province, P. R. China, 36°53'35"N, 102°25'56"E, elevation 2610m, 22 July 2007, coll. Xiao-Feng Xue.

##### Host.

*Picea asperata* Mast. (Pinaceae); *Picea meyeri* Rehd. Et Wils. (Pinaceae)

##### Relation to host.

Vagrant on leaf surface. No damage to the host was observed.

##### Distribution.

China (Shaanxi, Qinghai).

#### Phyllocoptes dangchangi

Song, Xue & Hong, 2006

http://species-id.net/wiki/Phyllocoptes_dangchangi

Phyllocoptes dangchangi Song et al. 2006: 38–40, figure 3.
Phyllocoptes dangchangi ; Song et al. 2008: 29.
Phyllocoptes dangchangi ; Xue et al. 2009: 134.
Phyllocoptes dangchangi ; Song et al. 2009c: 36.

##### Material examined.

11 females and 1 male (slide number NJAUEri811), from *Picea* sp. (Pinaceae), Beishan National Forest Park, Huzhu County, Qinghai Province, P. R. China, 36°53'35"N, 102°25'56"E, elevation 2610m, 22 July 2007, coll. Xiao-Feng Xue.

##### Host.

*Picea asperata* Mast. (Pinaceae); *Picea* sp. (Pinaceae).

##### Relation to host.

Vagrant on terminal part of the needles. No damage to the host was observed.

##### Distribution.

China (Gansu, Qinghai).

#### Phyllocoptes gansuensis

Kuang & Luo, 1998

http://species-id.net/wiki/Phyllocoptes_gansuensis

Phyllocoptes gansuensis Kuang et al. 1998: 201–203, figures 25–29.
Phyllocoptes gansuensis ; Huang 2001: 45.
Phyllocoptes gansunensis ; Kuang et al. 2005: 66–67, figure 64.
Phyllocoptes gansuensis ; Song et al. 2008: 30.
Phyllocoptes gansuensis ; Xue et al. 2009: 134.
Phyllocoptes gansuensis ; Song et al. 2009c: 37.

##### Material examined.

8 females and 2 males (slide number NJAUEri824), from a new host, *Potentilla parvifolia* Fisch. ap. Lehm. (Rosaceae), Beishan National Forest Park, Huzhu County, Qinghai Province, P. R. China, 36°53'35"N, 102°25'56"E, elevation 2610m, 22 July 2007, coll. Xiao-Feng Xue.

##### Host.

*Potentilla glabra* Lodd. (Rosaceae); *Potentilla parvifolica* Fisch. et. Lehm. (Rosaceae).

##### Relation to host.

Vagrant on leaf lower surface. No damage to the host was observed.

##### Distribution.

China (Gansu, Qinghai).

### Genus *Phyllocoptruta* Keifer, 1938

#### Phyllocoptruta platyclada

Xue, Song, Amrine & Hong, 2007

http://species-id.net/wiki/Phyllocoptruta_platyclada

Phyllocoptruta platyclada Xue et al. 2007: 340–342, figure 3.
Phyllocoptruta platyclada ; Xue et al. 2010: 698.

##### Material examined.

8 females (slide number NJAUEri814), from a new host, *Juniperus chinensis* L. (Cupressaceae), Beishan National Forest Park, Huzhu County, Qinghai Province, P. R. China, 36°53'35"N, 102°25'56"E, elevation 2610m, 22 July 2007, coll. Xiao-Feng Xue.

##### Host.

*Platycladus orientalis* (Linn.) Franco (Cupressaceae); *Juniperus chinensis* L. (Cupressaceae).

##### Relation to host.

Vagrant on terminal part of the needles. No damage to the host was observed.

##### Distribution.

China (Shaanxi, Qinghai).

### Tribe Anthocoptini Amrine & Stasny, 1994. Genus *Aculus* Keifer, 1959

#### Aculus changbais

Xue, Song & Hong, 2008

http://species-id.net/wiki/Aculus_changbais

Aculus changbais Xue et al. 2008: 41–42, figure 3.
Aculus changbais ; Song et al. 2009b: 3.

##### Material examined.

8 females and 1 male (slide number NJAUEri778), from a new host, *Salix chaenomeloides* Kimura (Salicaceae), Mengda Natural Reserve, Xunhua County, Qinghai Province, P. R. China, 35°47'38"N, 102°40'40"E, elevation 2523m, 19 July 2007, coll. Xiao-Feng Xue.

##### Host.

*Salix gracilistyla* Miq. (Salicaceae); *Salix chaenomeloides* Kimura (Salicaceae).

##### Relation to host.

Vagrant on leaf lower surface. No damage to the host was observed.

##### Distribution.

China (Jilin, Qinghai).

#### Aculus huangzhongensis

Kuang, 2000

http://species-id.net/wiki/Aculus_huangzhongensis

Aculus huangzhongensis Kuang 2000: 392–393, figures 13–18.
Aculus huangzhongensis ; Kuang et al. 2005: 91–92, figure 91.
Aculus huangzhongensis ; Song et al. 2009b: 2.

##### Host.

*Syringa oblata* Lindl. (Oleaceae).

##### Relation to host.

Vagrant on leaf surface. No damage to the host was observed.

##### Distribution.

China (Qinghai).

### Genus *Aculodes* Keifer, 1966

#### Aculodes salicis

Kuang, 1997

http://species-id.net/wiki/Aculodes_salicis

Aculodes salicis Kuang 1997: 232–233, figures 12–15.
Aculodes salicis ; Skoracka 2003: 43.
Aculodes salicis ; Kuang et al. 2005: 80–81, figure 79.

##### Host.

*Salix babylonica* L. (Salicaceae).

##### Relation to host.

Forming galls on the leaf of the host.

##### Distribution.

China (Qinghai), Poland.

### Genus *Aculops* Keifer, 1966

#### Aculops ulmi

Hong & Xue, 2005

http://species-id.net/wiki/Aculops_ulmi

**Figure 12.**
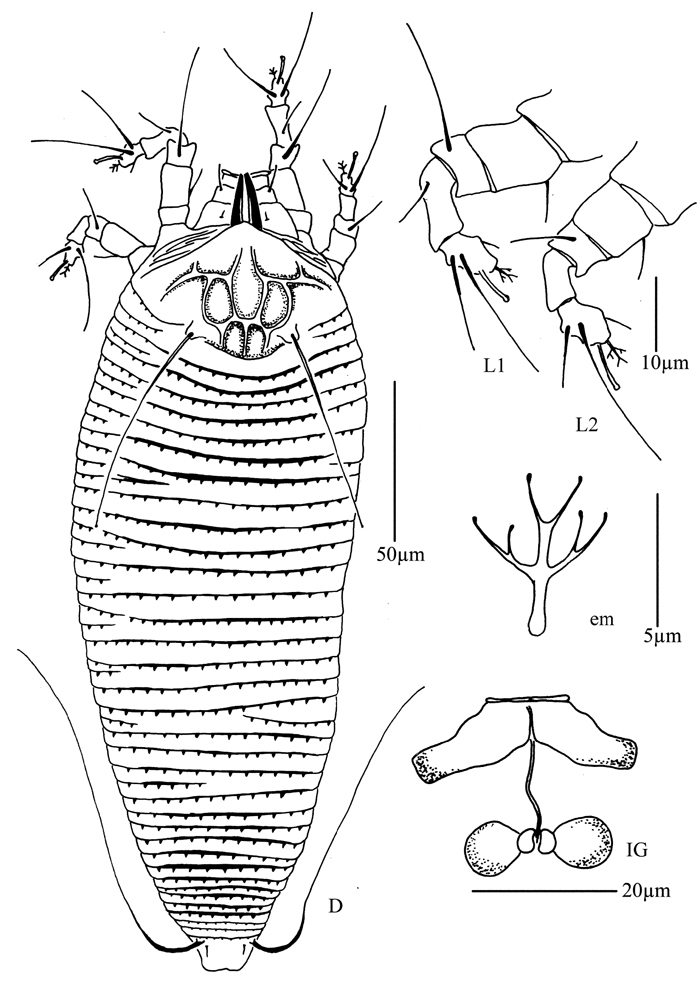
*Aculops ulmi* Hong & Xue: **D** dorsal view of female **L1** leg I **L2** leg II **em** empodium **IG**  female internal genitalia.

Figures 12 [Fig F13] [Fig F14] –15

Aculops ulmi Hong and Xue 2005: 205, 209.

##### Redescription.

Female. (n = 10) Body fusiform, light yellow, 192 (192–230), 70 (62–72) wide, 80 (80–81) thick. **Gnathosoma** 23 (23–25), projecting obliquely down, suboral plate present, pedipalp coxal seta (*ep*) 3 (2–4), dorsal pedipalp genual seta (*d*) 6 (5–6), cheliceral stylets 22 (22–25). **Prodorsal shield** 33 (33–34), 48 (48–51) wide, subtriangular; median, admedian and submedian lines present, median line ending at basal 1/4 of prodorsal shield, median and admedian lines connected at basal 1/4 of prodorsal shield, admedian and submedian lines connected at basal 2/3 of prodorsal shield, forming two cells on both sides of median line. Scapular tubercles on rear shield margin, 4 (4–5), 26 (26–28) apart, scapular setae (*sc*) 55 (55–60), projecting posteriorly, knobbed at the end. **Coxigenital region** with 7 (6–7) annuli, with triangular microtubercles. Coxisternal plates with short lines and granules, anterolateral setae on coxisternum **I** (*1b*) 13 (10–13), 14 (14–15) apart, proximal setae on coxisternum **I** (*1a*) 30 (27–30), 11 (11–13) apart, proximal setae on coxisternum **II** (*2a*) 54 (54–57), 26 (26–28) apart, tubercles *1b* and *1a* 7 (7–8) apart, tubercles *1a* and *2a* 9 (9–10) apart. Prosternal apodeme combined, 7 (5–11). **Leg I** 31 (31–35), femur 10 (9–12), basiventral femoral seta (*bv*) 12 (11–14); genu 5 (5–6), antaxial genual seta (*l"*) 22 (22–29); tibia 7 (7–9), paraxial tibial seta (*l’*) 6 (6–8), located at 1/3 from dorsal base; tarsus 8 (8–11), seta *ft’* 16 (16–18), seta *ft"* 20 (20–25), seta *u’* 6 (5–6); tarsal empodium (*em*) 6 (6–7), simple, 2-rayed, tarsal solenidion (*ω*) 9 (8–10), slightly knobbed. **Leg II** 28 (28–31), femur 9 (9–11), basiventral femoral seta (*bv*) 12 (11–12); genu 5 (5–6), antaxial genual seta (*l"*) 9 (9–13); tibia 5 (5–7); tarsus 9 (9–10), seta *ft’* 7 (6–7), seta *ft"* 18 (18–20), seta *u’* 5 (4–5); tarsal empodium (*em*) 6 (6–7), simple, 2-rayed, tarsal solenidion (*ω*) 8 (8–11), slightly knobbed. **Opisthosoma** dorsally with 35 (22–38) annuli, with triangular microtubercles, ventrally with 55 (55–56) annuli, with triangular microtubercles. Setae *c2* 16 (14–16) on ventral annulus 10 (10–11), 61 (61–69) apart; setae *d* 57 (55–65) on ventral annulus 21 (21–22), 50 (48–50) apart; setae *e* 14 (12–19) on ventral annulus 33 (32–34), 23 (23–24) apart; setae *f* 27 (26–30) on ventral annulus 51 (49–53), 21 (19–21) apart. Setae *h1* 3 (3–4), *h2* 90 (85–90). **Female genitalia** 11 (11–14), 23 (22–23) wide, coverflap with 8 longitudinal ridges, setae *3a* 17 (17–22), 17 (16–17) apart.

Male. Unknown.

**Figure 13. F13:**
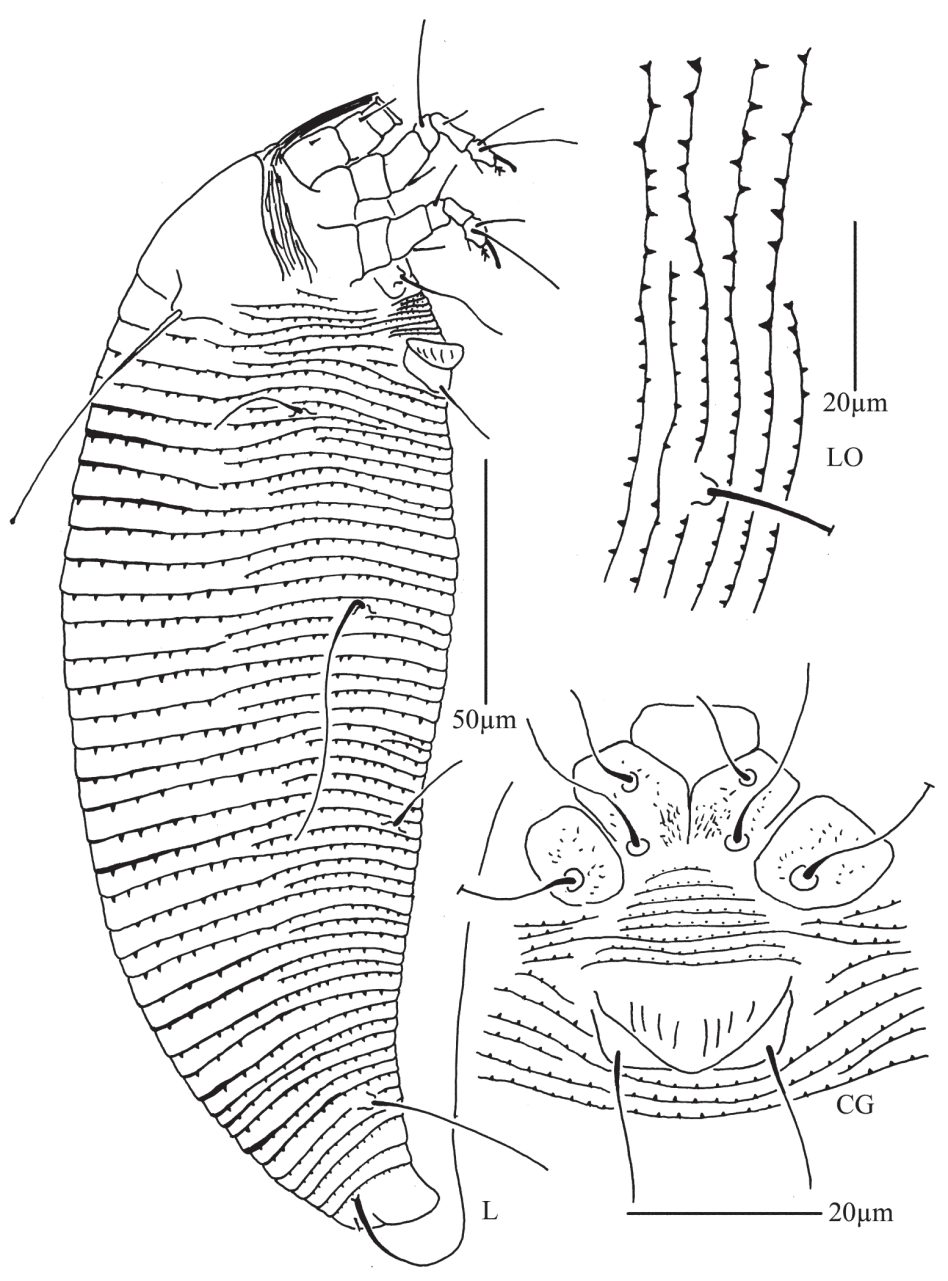
*Aculops ulmi* Hong & Xue: **L** lateral view of female **LO** lateral microtubercles **CG** coxae and female genitalia.

**Figure 14. F14:**
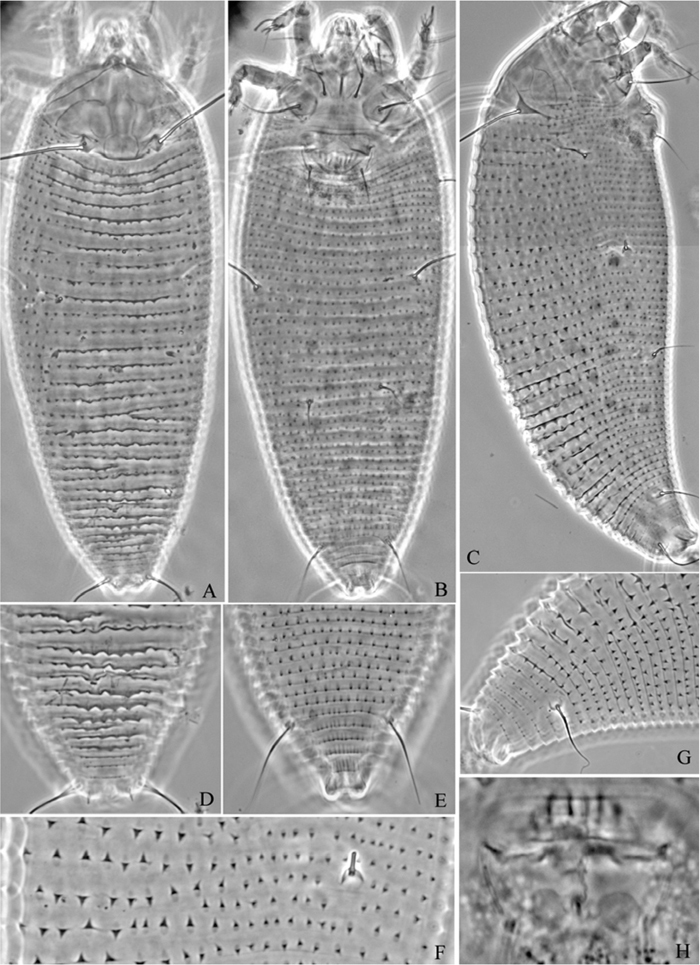
*Aculops ulmi* Hong & Xue: **A** dorsal view of female **B** ventral view of female **C** lateral view of female **D** dorsal view of female posterior part **E** ventral view of female posterior part **F** lateral microtubercles **G** lateral view of female posterior part **H** female internal genitalia.

**Figure 15.**
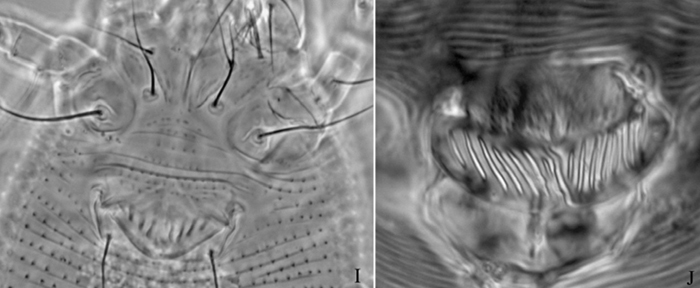
*Aculops ulmi* Hong & Xue: **I** female genitalia **J** female genitalia from holotype.

##### Type material.

Hong and Xue (2005) described types as follows: Holotype female (slide number 17.viii.2003), from *Ulmus* sp. (Ulmaceae), Xingtai city, Hebei Province, P. R. China, coll. Xiao-Feng Xue. Paratypes (slide number 17.2003). 9 females and 2 males. All types here were re-examined. Female genitalia coverflap with 10–12 longitudinal ridges.

##### Additional material.

6 females (slide number NJAUEri792) from *Ulmus* sp. (Ulmaceae), Xining, Qinghai Province, P. R. China, 36°38'18"N, 101°45'27"E, elevation 2241m, 21 July 2007, coll. Xiao-Feng Xue.

##### Relation to host.

Vagrant on leaf lower surface. No damage to the host was observed.

##### Distribution.

China (Hebei, Qinghai).

##### Notes.

Instead of the original description: female genitalia coverflap smooth, female genitalia coverflap is with 8 longitudinal ridges in this redescription.

#### Aculops xiningensis

Kuang, 2000

http://species-id.net/wiki/Aculops_xiningensis

Aculops xiningensis Kuang 2000: 392, figures 7–12.
Aculops xiningensis ; Kuang et al. 2005: 86–87, figure 86.

##### Host.

*Malus pumila* P. Mill. (Rosaceae).

##### Relation to host.

Vagrant on leaf lower surface. No damage to the host was observed.

##### Distribution.

China (Qinghai).

### Genus *Tetraspinus* Boczek, 1961

#### Tetraspinus syringae

Lin & Kuang, 2001

http://species-id.net/wiki/Tetraspinus_syringae

Tetraspinus syringae Lin and Kuang 2001: 351–353, figures 11–16.
Tetraspinus syringae ; Kuang et al. 2005: 125–126, figure 128.

##### Host.

*Syringa oblata* Lindl. (Oleaceae).

##### Relation to host.

Vagrant on leaf lower surface. No damage to the host was observed.

##### Distribution.

China (Qinghai).

### Genus *Tetra* Keifer, 1944

#### Tetra pinnatifidae

Xue, Song & Hong, 2006

http://species-id.net/wiki/Tetra_pinnatifidae

Tetra pinnatifidae Xue et al. 2006a: 6–8, figure 2.

##### Material examined.

8 females and 2 males (slide number NJAUEri793) from a new host, *Prunus armeniaca* Linn. (Rosaceae), Xining, Qinghai Province, P. R. China, 36°38'18"N, 101°45'27"E, elevation 2241m, 21 July 2007, coll. Xiao-Feng Xue.

##### Host. 

*Crataegus pinnatifida* Bunge (Rosaceae); *Prunus armeniaca* Linn. (Rosaceae).

##### Relation to host.

Vagrant on leaf lower surface. No damage to the host was observed.

##### Distribution.

China (Shaanxi, Qinghai).

#### Tetra pruniana sp. n.

urn:lsid:zoobank.org:act:D0B42704-F828-4F47-9265-47B3EF8E603A

http://species-id.net/wiki/Tetra_pruniana

**Figure 16.**
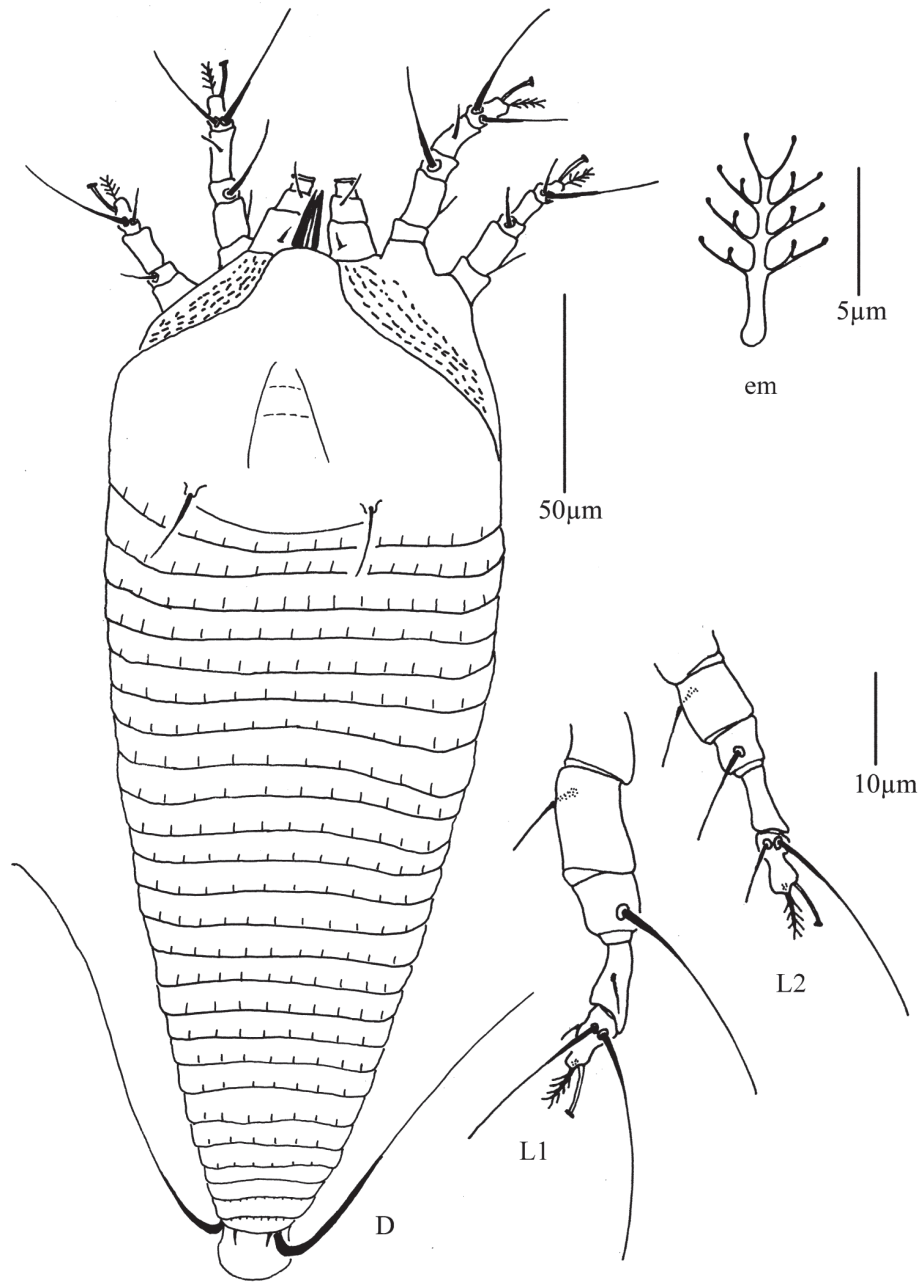
*Tetra pruniana* sp. n.: **D** dorsal view of female **em** empodium **L1** leg I **L2** leg II.

Figures 16 [Fig F17] –18

##### Description.

Female. (n = 9) Body fusiform, light yellow, 215 (210–225), 77 (75–78) wide. **Gnathosoma** 21 (21–23), projecting obliquely down, suboral plate present, pedipalp coxal seta (*ep*) 4 (4–5), dorsal pedipalp genual seta (*d*) 7 (7–8), cheliceral stylets 16 (16–18). **Prodorsal shield** 54 (54–55), 75 (75–78) wide, subtriangular; frontal lobe 12 (11–12); median and submedian lines absent, admedian lines connected by two weak transverse lines. Scapular tubercles near rear shield margin, 3 (3–4), 34 (32–34) apart, scapular setae (*sc*) 14 (13–15), projecting posteriorly. Rear shield with wave-like margin. **Coxigenital region** with 9 annuli. Coxisternal plates with short lines, anterolateral setae on coxisternum **I** (*1b*) 11 (10–13), 14 (13–14) apart, proximal setae on coxisternum **I** (*1a*) 20 (20–25), 10 (10–11) apart, proximal setae on coxisternum **II** (*2a*) 50 (45–50), 28 (28–29) apart, tubercles *1b* and *1a* 7 (7–8) apart, tubercles *1a* and *2a* 10 (10–12) apart. Prosternal apodeme combined, 8 (6–8). **Leg I** 34 (32–35), femur 12 (12–13), basiventral femoral seta (*bv*) 12 (12–14); genu 7 (6–7), antaxial genual seta (*l"*) 20 (19–20); tibia 10 (9–10), paraxial tibial seta (*l’*) 6 (5–6), located at 1/3 from dorsal base; tarsus 7 (7–8), seta *ft’* 19 (19–21), seta *ft"* 25 (24–25), seta *u’* 5 (4–5); tarsal empodium (*em*) 7 (6–7), simple, 4-rayed, tarsal solenidion (*ω*) 6 (6–7), knobbed. **Leg II** 27 (27–31), femur 10 (10–11), basiventral femoral seta (*bv*) 12 (12–13); genu 5 (5–6), antaxial genual seta (*l"*) 8 (7–8); tibia 6 (6–7); tarsus 6 (6–7), seta *ft’* 6 (6–7), seta *ft"* 25 (23–25), seta *u’* 5 (5–6); tarsal empodium (*em*) 6 (5–6), simple, 4-rayed, tarsal solenidion (*ω*) 6 (6–7), knobbed. **Opisthosoma** dorsally with 25 (24–25) annuli, with weak filamentous microtubercles, ventrally with 53 (53–59) annuli, with round microtubercles. Setae *c2* 25 (24–26) on ventral annulus 9 (9–11), 58 (58–59) apart; setae *d* 60 (57–65) on ventral annulus 22 (22–24), 33 (32–33) apart; setae *e* 18 (17–20) on ventral annulus 37 (37–40), 18 (17–18) apart; setae *f* 29 (29–33) on ventral annulus 50 (50–56), 25 (22–25) apart. Setae *h1* 3 (2–3), *h2* 85 (80–95). **Female genitalia** 15 (15–17), 25 (24–25) wide, coverflap with 10 longitudinal ridges, setae *3a* 18 (16–19), 17 (17–18) apart.

Male. (n = 6) Body fusiform, light yellow, 170–186, 73–74 wide. **Gnathosoma** 16–17, projecting obliquely downwards, suboral plate present, pedipalp coxal seta (*ep*) 4–5, dorsal pedipalp genual seta (*d*) 7–8, cheliceral stylets 14–16. **Prodorsal shield** has the same design as female, 47–50, 72–73 wide, subtriangular; frontal lobe 10–12. Scapular tubercles on rear shield margin, 3–4, 30–33 apart, scapular setae (*sc*) 7–9, projecting posteriorly. **Coxigenital region** with 9 smooth annuli. Coxisternal plates with short lines, anterolateral setae on coxisternum **I** (*1b*) 10–11, 13–14 apart, proximal setae on coxisternum **I** (*1a*) 21–22, 10–11 apart, proximal setae on coxisternum **II** (*2a*) 45–48, 28–30 apart, tubercles *1b* and *1a* 7–8 apart, tubercles *1a* and *2a* 9–10 apart. Prosternal apodeme combined, 7–8. **Leg I** 34–35, femur 10–12, basiventral femoral seta (*bv*) 9–10; genu 6–7, antaxial genual seta (*l"*) 19–22; tibia 9–10, paraxial tibial seta (*l’*) 6–7, located at 1/3 from dorsal base; tarsus 7–8, seta *ft’* 15–17, seta *ft" 21*–24, seta *u’* 4–5; tarsal empodium (*em*) 7–8, simple, 4-rayed, tarsal solenidion (*ω*) 6–7, knobbed. **Leg II** 28–29, femur 9–10, basiventral femoral seta (*bv*) 10–11; genu 5–6, antaxial genual seta (*l"*) 6–7; tibia 7–8; tarsus 6–7, seta *ft’* 6–7, seta *ft"* 22–25, seta *u’* 5–6; tarsal empodium (*em*) 6–7, simple, 4-rayed, tarsal solenidion (*ω*) 7–8, knobbed. **Opisthosoma** dorsally with 26–27 annuli, with weak filamentous microtubercles, ventrally with 53–54 annuli, with round microtubercles. Setae *c2* 18–20 on ventral annulus 10–11, 55–65 apart; setae *d* 32–35 on ventral annulus 20–22, 30–45 apart; setae *e* 18–20 on ventral annulus 33–35, 18–30 apart; setae *f* 25–28 on ventral annulus 50–51, 20–22 apart. Setae *h1* 2–3, *h2* 80–90. **Male genitalia** 13–14, 23–28 wide, setae *3a* 17–18, 20–21 apart.

**Figure 17. F17:**
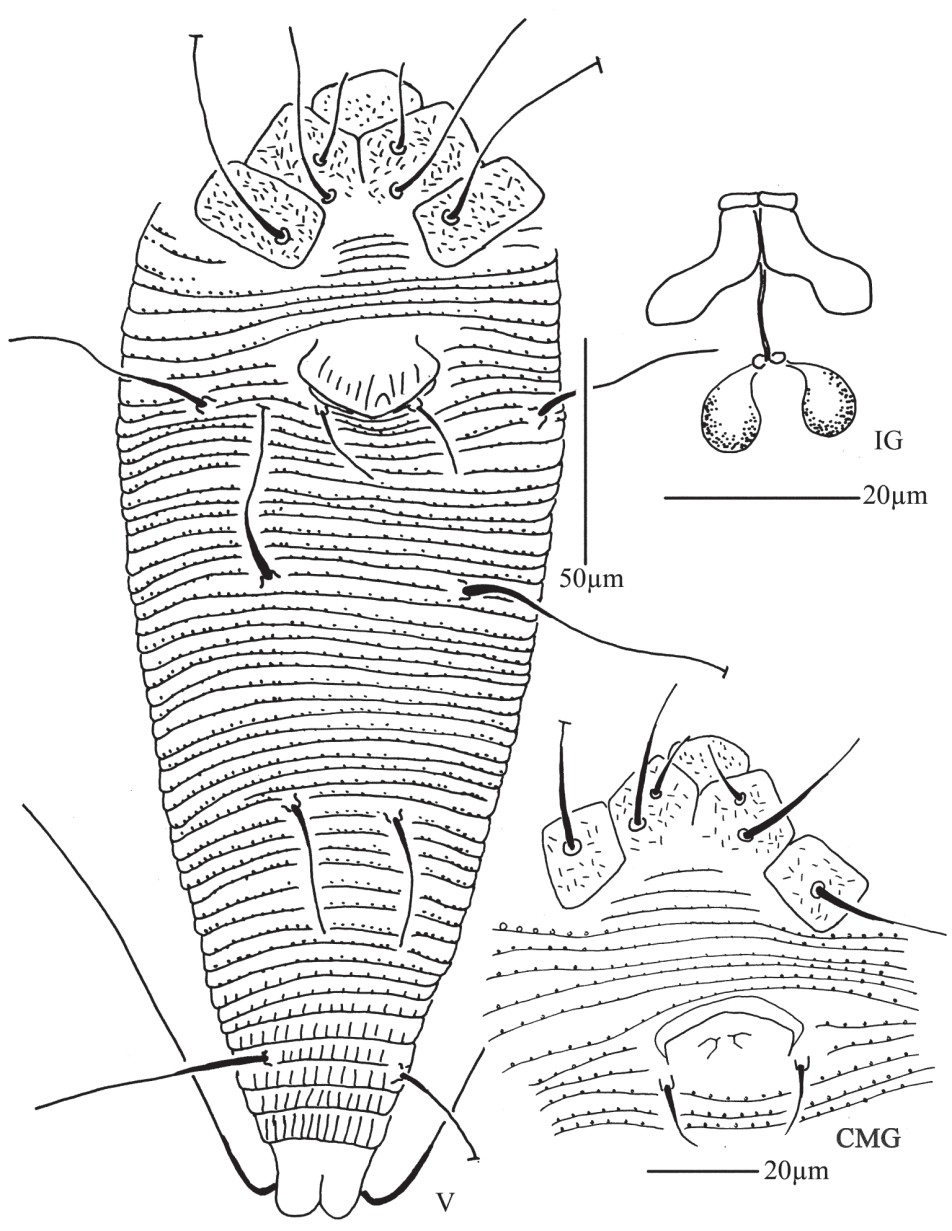
*Tetra pruniana* sp. n.: **V** ventral view of female **IG** female internal genitalia **CMG** coxae and male genitalia.

**Figure 18.**
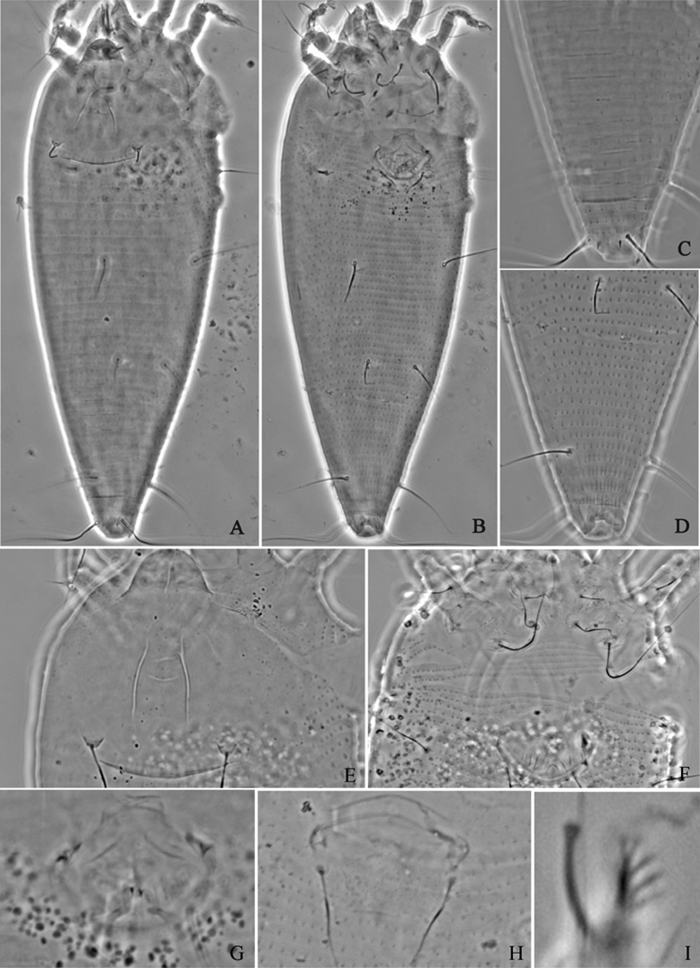
*Tetra pruniana*sp. n.: **A** dorsal view of female **B** ventral view of female **C** dorsal view of female posterior part **D** ventral view of female posterior part **E** prodorsal shield **F** coxae and female genitalia **G** female internal genitalia **H** male genitalia **I** empodium.

##### Type material.

**Holotype**, female (slide number NJAUEri808, marked Holotype), from *Prunus tomentosa* Thunb. (Rosaceae), Xining City, Qinghai Province, P. R. China, 36°38'18"N, 101°45'27"E, elevation 2241m, 21 July 2007, coll. Xiao-Feng Xue. **Paratypes**, 2 females and 4 males (slide number NJAUEri808), with the same data as holotype.

##### Relation to host.

Vagrant on leaf lower surface. No damage to the host was observed.

##### Etymology.

The specific designation *pruniana* is from the generic name of host plant, *Prunus*; feminine in gender.

##### Differential diagnosis.

This species is similar to *Tetra pinnatifidae* Xue, Song & Hong, 2006a, but can be differentiated from the latter by median and submedian lines absent (median and submedian lines present in *Tetra pinnatifidae*), admedian lines connected by two weak transverse lines (admedian lines separated in *Tetra pinnatifidae*).

#### Tetra pyriana sp. n.

urn:lsid:zoobank.org:act:99B1B2A9-DB3F-466C-8900-951BB3B35485

http://species-id.net/wiki/Tetra_pyriana

**Figure 19.**
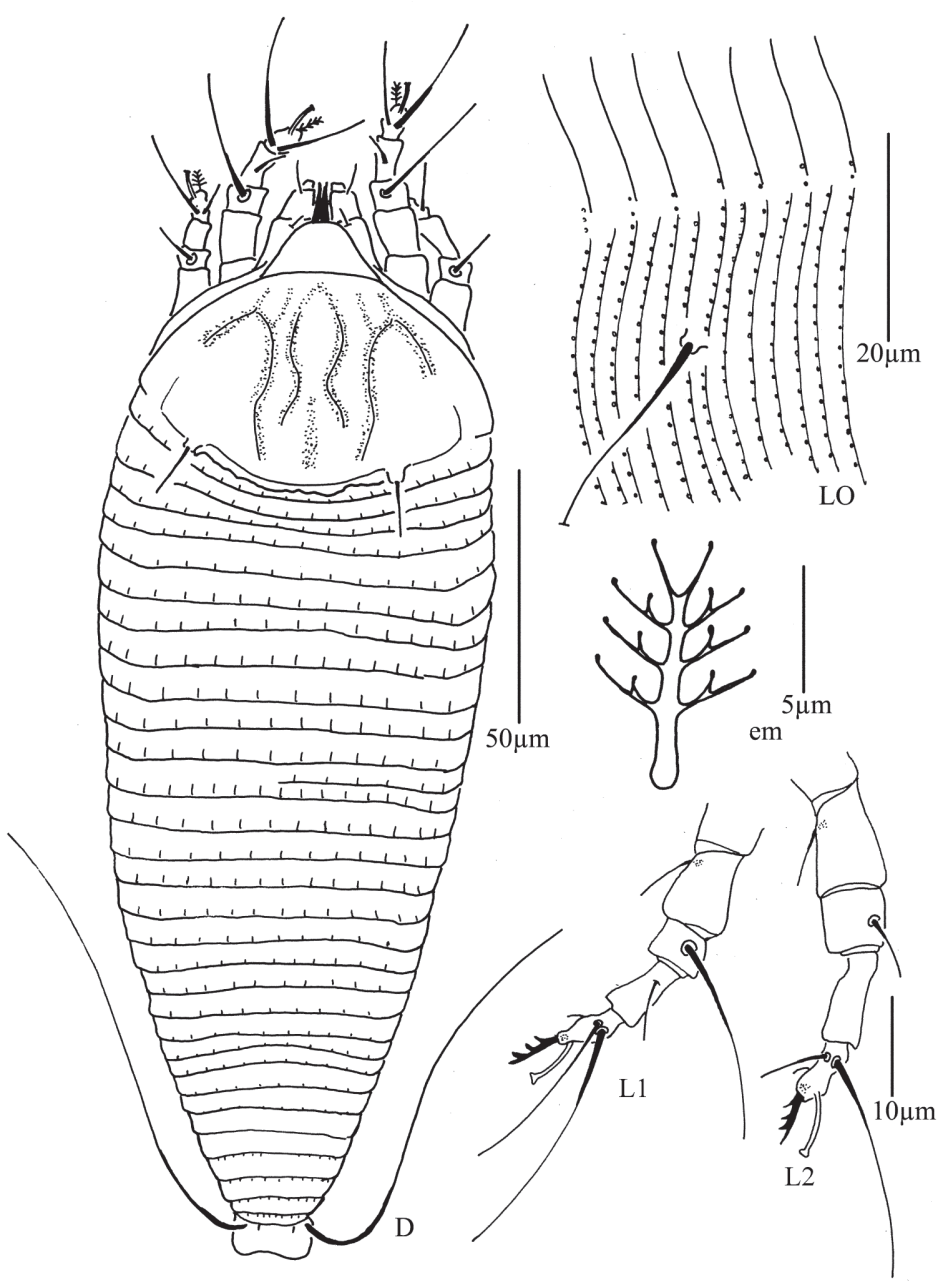
*Tetra pyriana* sp. n.: **D** dorsal view of female **LO** lateral microtubercles **em** empodium **L1** leg I **L2** leg II.

Figures 19 [Fig F20] [Fig F21] –22

##### Description.

Female. (n = 11) Body fusiform, light yellow, 180 (169–185), 73 (72–76) wide, 65 (64–65) thick. **Gnathosoma** 20 (20–23), projecting obliquely down, pedipalp coxal seta (*ep*) 4 (3–4), dorsal pedipalp genual seta (*d*) 6 (5–7), cheliceral stylets 20 (20–21). **Prodorsal shield** 47 (47–49), 68 (68–72) wide, subtriangular; frontal lobe 12 (11–13); median, admedian and submedian lines present, median line obscure and ending at basal 1/3 of prodorsal shield. Scapular tubercles on rear shield margin, 4 (3–4), 36 (33–36) apart, scapular setae (*sc*) 12 (12–13), projecting posteriorly. **Coxigenital region** with 13 (12–13) smooth annuli. Coxisternal plates with short lines, anterolateral setae on coxisternum **I** (*1b*) 7 (7–9), 12 (11–12) apart, proximal setae on coxisternum **I** (*1a*) 21 (20–21), 10 (9–10) apart, proximal setae on coxisternum **II** (*2a*) 40 (40–45), 27 (25–27) apart, tubercles *1b* and *1a* 6 (6–7) apart, tubercles *1a* and *2a* 10 (9–10) apart. Prosternal apodeme combined, 7 (6–7). **Leg I** 32 (30–32), femur 12 (11–12), basiventral femoral seta (*bv*) 11 (10–11); genu 6 (5–6), antaxial genual seta (*l"*) 20 (20–21); tibia 7 (7–8), paraxial tibial seta (*l’*) 5 (5–6), located at 1/3 from dorsal base; tarsus 8 (7–8), seta *ft’* 19 (17–19), seta *ft"* 25 (21–25), seta *u’* 5 (4–5); tarsal empodium (*em*) 6 (6–7), simple, 4-rayed, tarsal solenidion (*ω*) 6 (6–7), knobbed. **Leg II** 28 (27–28), femur 11 (10–11), basiventral femoral seta (*bv*) 8 (8–11); genu 5 (4–5), antaxial genual seta (*l"*) 8 (7–8); tibia 7 (6–7); tarsus 7 (6–7), seta *ft’* 5 (5–6), seta *ft"* 20 (19–20), seta *u’* 4 (4–5); tarsal empodium (*em*) 6 (6–7), simple, 4-rayed, tarsal solenidion (*ω*) 5 (5–6), knobbed. **Opisthosoma** dorsally with 32 (30–32) annuli, with filamentous microtubercles, ventrally with 58 (58–60) annuli, with round microtubercles. Setae *c2* 25 (24–30) on ventral annulus 11 (11–13), 57 (54–57) apart; setae *d* 45 (45–50) on ventral annulus 22 (22–24), 32 (32–33) apart; setae *e* 18 (17–18) on ventral annulus 37 (37–41), 17 (16–17) apart; setae *f* 29 (28–30) on ventral annulus 53 (53–54), 25 (24–25) apart. Setae *h1* 3 (2–3), *h2* 85 (80–90). **Female genitalia** 15 (14–16), 22 (22–23) wide, coverflap with 11 (10–12) longitudinal ridges, setae *3a* 22 (19–24), 14 (14–15) apart.

Male. (n = 2) Body fusiform, light yellow, 150–176 63–65 wide. **Gnathosoma** 21–22, projecting obliquely down, pedipalp coxal seta (*ep*) 2–3, dorsal pedipalp genual seta (*d*) 6–7, cheliceral stylets 19–20. **Prodorsal shield** has the same design as female, 42–47, 63–65 wide, subtriangular; frontal lobe 10–11. Scapular tubercles on rear shield margin, 3–4, 30–31 apart, scapular setae (*sc*) 11–13, projecting posteriorly. **Coxigenital region** with 12–13 smooth annuli. Coxisternal plates with short lines, anterolateral setae on coxisternum **I** (*1b*) 7–8, 12–13 apart, proximal setae on coxisternum **I** (*1a*) 18–20, 9–10 apart, proximal setae on coxisternum **II** (*2a*) 45–46, 24–26 apart, tubercles *1b* and *1a* 6–7 apart, tubercles *1a* and *2a* 7–9 apart. Prosternal apodeme combined, 5–6. **Leg I** 29–32, femur 7–9, basiventral femoral seta (*bv*) 9–12; genu 5–6, antaxial genual seta (*l"*) 20–23; tibia 6–8, paraxial tibial seta (*l’*) 5–6, located at 1/3 from dorsal base; tarsus 7–8, seta *ft’* 17–18, seta *ft"* 21–23, seta *u’* 3–4; tarsal empodium (*em*) 6–7, simple, 4-rayed, tarsal solenidion (*ω*) 5–6, knobbed. **Leg II** 25–27, femur 7–9, basiventral femoral seta (*bv*) 9–10; genu 4–5, antaxial genual seta (*l"*) 6–8; tibia 7–8; tarsus 6–7, seta *ft’* 2–3, seta *ft"* 20–21, seta *u’* 2–3; tarsal empodium (*em*) 6–7, simple, 4-rayed, tarsal solenidion (*ω*) 6–7, knobbed. **Opisthosoma** dorsally with 29–31 annuli, with filamentous microtubercles, ventrally with 60–61 annuli, with round microtubercles. Setae *c2* 24–25 on ventral annulus 13–14, 50–51 apart; setae *d* 36–37 on ventral annulus 24–25, 29–30 apart; setae *e* 15–16 on ventral annulus 41–42, 16–17 apart; setae *f* 28–29 on ventral annulus 55–56, 20–21 apart. Setae *h1* 2–3, *h2* 85–90. **Male genitalia** 13–14, 19–20 wide, setae *3a* 17–18, 16–17 apart.

**Figure 20. F20:**
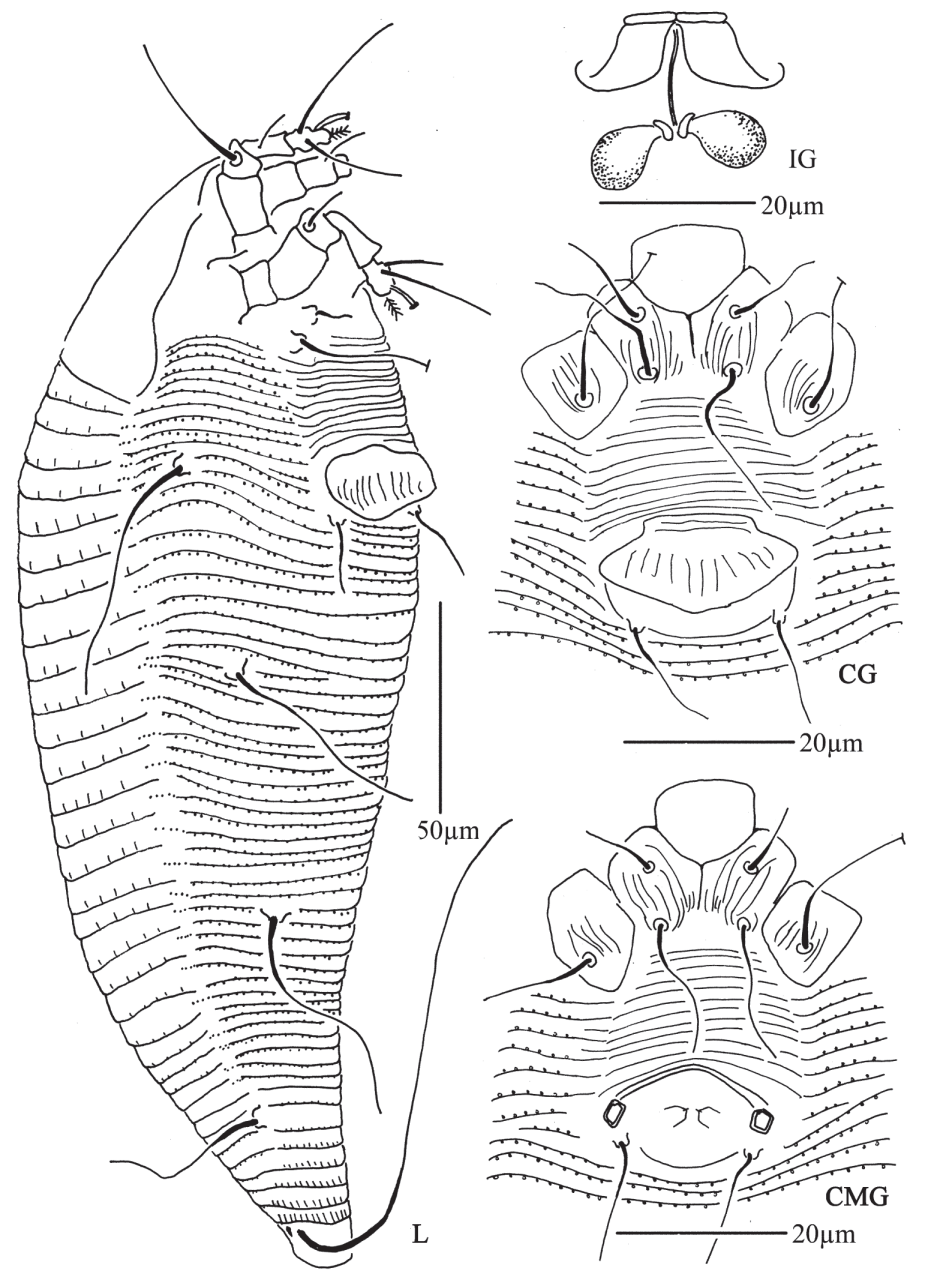
*Tetra pyriana* sp. n.: **L** lateral view of female **IG** female internal genitalia **CG** coxae and female genitalia **CMG** coxae and male genitalia.

**Figure 21. F21:**
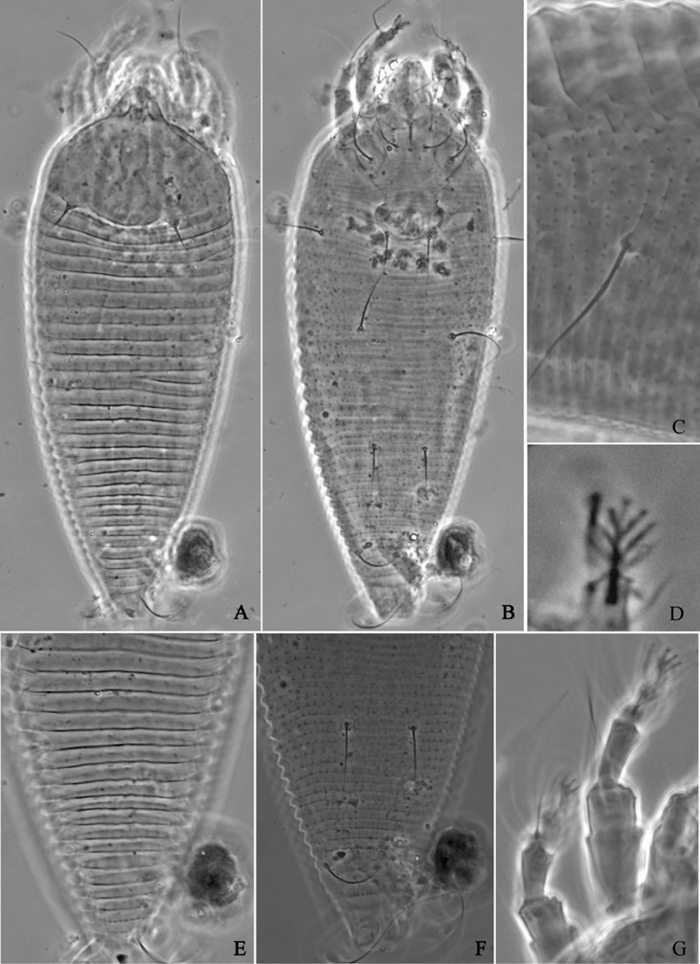
*Tetra pyriana* sp. n.: **A** dorsal view of female **B** ventral view of female **C** lateral microtubercles **D  **empodium **E** dorsal view of female posterior part **F** ventral view of female posterior part **G** leg I and leg II.

**Figure 22.**
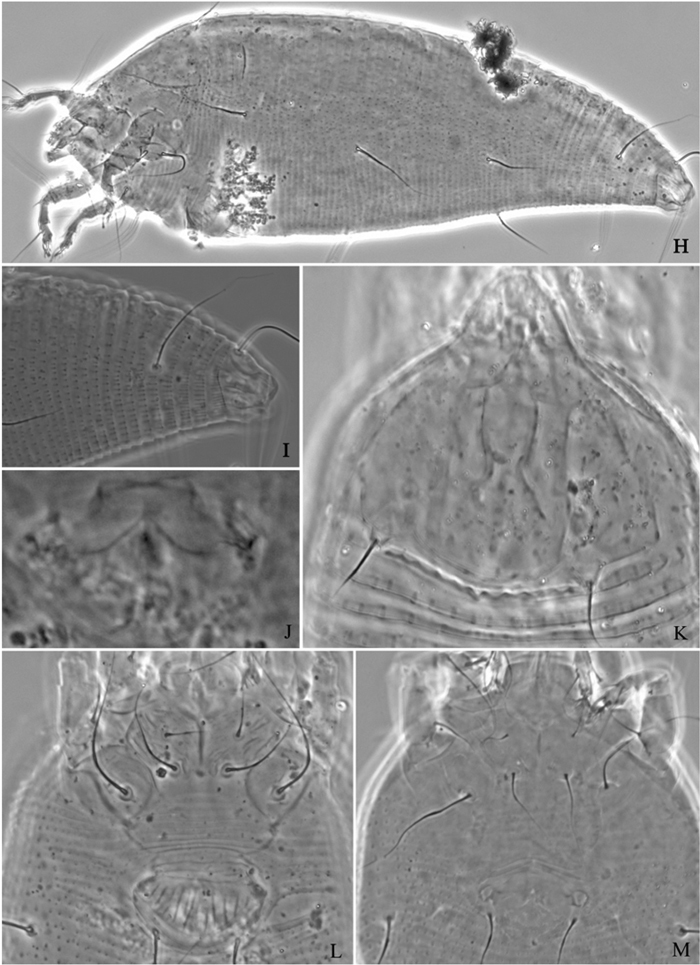
*Tetra pyriana* sp. n.: **H** lateral view of female **I** lateral view of female posterior part **J** female internal genitalia **K** prodorsal shield **L** coxae and female genitalia **M** coxae and male genitalia.

##### Type material.

**Holotype**, female (slide number NJAUEri796, marked Holotype), from *Pyrus calleryana* Decne. (Rosaceae), Xining, Qinghai Province, P. R. China, 36°38'18"N, 101°45'27"E, elevation 2241m, 21 July 2007, coll. Xiao-Feng Xue. **Paratypes**, 10 females and 2 males (slide number NJAUEri796), with the same data as holotype.

##### Additional material.

13 females (slide number NJAUEri807), from *Pyrus betulifolia* Bunge. (Rosaceae), Xining, Qinghai Province, P. R. China, 36°38'18"N, 101°45'27"E, elevation 2241m, 21 July 2007, coll. Xiao-Feng Xue; 14 females and 1 male (slide number NJAUEri806), from *Pyrus calleryana* Decne. (Rosaceae), Xining, Qinghai Province, P. R. China, 36°38'18"N, 101°45'27"E, elevation 2241m, 21 July 2007, coll. Xiao-Feng Xue.

##### Relation to host.

Vagrant on leaf lower surface. No damage to the host was observed.

##### Etymology.

The specific designation *pyriana* is from the generic name of host plant, *Pyrus*; feminine in gender.

##### Differential diagnosis.

This species is similar to *Tetra pinnatifidae* Xue, Song & Hong, 2006a, but can be differentiated from the latter by median line weak and ending at basal 1/3 of prodorsal shield (median normal and does not disappear at basal 1/3 of prodorsal shield in *Tetra pinnatifidae*), coxigenital region with smooth annuli (coxigenital region annuli with round microtubercles in *Tetra pinnatifidae*).

#### Tetra simonia sp. n.

urn:lsid:zoobank.org:act:11D2D601-440B-4A7D-AD50-18F7B91B3A33

http://species-id.net/wiki/Tetra_simonia

**Figure 23.**
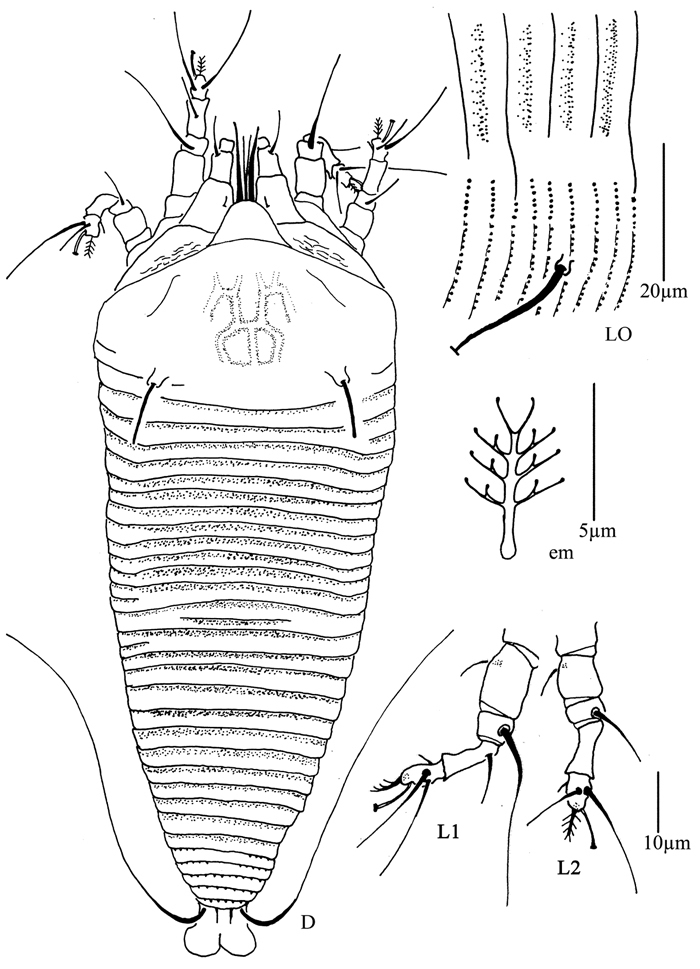
*Tetra simonia* sp. n.: **D** dorsal view of female **LO** lateral microtubercles **em** empodium **L1** leg I **L2** leg II.

Figures 23 [Fig F24] [Fig F25] –26

##### Description.

Female. (n = 14) Body fusiform, light yellow, 196 (196–224), 72 (72–77) wide, 78 (78) thick. **Gnathosoma** 22 (20–22), projecting obliquely down, pedipalp coxal seta (*ep*) 3 (3–4), dorsal pedipalp genual seta (*d*) 12 (10–12), cheliceral stylets 19 (19–20). **Prodorsal shield** 46 (42–46), 72 (72–77) wide, subtriangular; frontal lobe 11 (11–12); median, admedian and submedian lines robust and connected, forming an “M” shape in the middle and two “H” shapes anterior to the “M”. Scapular tubercles on rear shield margin, 3 (3–4), 46 (44–46) apart, scapular setae (*sc*) 14 (14–15), projecting posteriorly. **Coxigenital region** with 14 (14–15) smooth annuli. Coxisternal plates smooth, anterolateral setae on coxisternum **I** (*1b*) 11 (11–12), 14 (14–19) apart, proximal setae on coxisternum **I** (*1a*) 17 (17–23), 12 (12–13) apart, proximal setae on coxisternum **II** (*2a*) 53 (53–58), 28 (27–31) apart, tubercles *1b* and *1a* 8 (8–9) apart, tubercles *1a* and *2a* 9 (9–10) apart. Prosternal apodeme combined, 6 (6–8). **Leg I** 34 (34–37), femur 12 (11–12), basiventral femoral seta (*bv*) 12 (12–13); genu 8 (8–9), antaxial genual seta (*l"*) 24 (22–24); tibia 13 (11–13), paraxial tibial seta (*l’*) 7 (6–7), located at 1/3 from dorsal base; tarsus 7 (7–8), seta *ft’* 22 (21–22), seta *ft"* 23 (22–23), seta *u’* 6 (6–7); tarsal empodium (*em*) 6 (6–7), simple, 4-rayed, tarsal solenidion (*ω*) 6 (6–7), knobbed. **Leg II** 30 (30–34), femur 11 (11–12), basiventral femoral seta (*bv*) 13 (12–13); genu 6 (6–7), antaxial genual seta (*l"*) 11 (11–13); tibia 9 (7–9); tarsus 7 (6–7), seta *ft’* 5 (5–6), seta *ft"* 22 (22–24), seta *u’* 6 (6–7); tarsal empodium (*em*) 6 (6–7), simple, 4-rayed, tarsal solenidion (*ω*) 6 (6–7), knobbed. **Opisthosoma** dorsally with 31 (30–31) annuli, with dark shading on rear annular margins, ventrally with 58 (58–65) annuli, with round microtubercles. Setae *c2* 36 (36–42) on ventral annulus 11 (11–13), 52 (52–56) apart; setae *d* 77 (71–77) on ventral annulus 21 (21–23), 33 (33–38) apart; setae *e* 23 (20–23) on ventral annulus 37 (37–39), 18 (18–19) apart; setae *f* 40 (36–40) on ventral annulus 51 (51–54), 30 (30–32) apart. Setae *h1* 5 (4–5), *h2* 150 (135–150). **Female genitalia** 20 (18–22), 25 (24–25) wide, coverflap with 10 (10–13) longitudinal ridges, setae *3a* 17 (17–18), 18 (17–18) apart.

Male. (n = 1) Body fusiform, light yellow, 188, 74 wide. **Gnathosoma** 21, projecting obliquely downwards, pedipalp coxal seta (*ep*) 3, dorsal pedipalp genual seta (*d*) 11, cheliceral stylets 19. **Prodorsal shield** has the same design as female, 42, 74 wide, subtriangular; frontal lobe 11. Scapular tubercles on rear shield margin, 44 apart, scapular setae (*sc*) 13, projecting posteriorly. **Coxigenital region** with 14 smooth annuli. Coxisternal plates smooth, anterolateral setae on coxisternum **I** (*1b*) 13), 14 apart, proximal setae on coxisternum **I** (*1a*) 23, 12 apart, proximal setae on coxisternum **II** (*2a*) 49, 30 apart, tubercles *1b* and *1a* apart 8, tubercles *1a* and *2a* 10 apart. Prosternal apodeme combined, 6. **Leg I** 35, femur 11, basiventral femoral seta (*bv*) 12; genu 8, antaxial genual seta (*l"*) 26; tibia 12, paraxial tibial seta (*l’*) 6, located at 1/3 from dorsal base; tarsus 7, seta *ft’* 21, seta *ft"* 24, seta *u’* 6; tarsal empodium (*em*) 6, simple, 4-rayed, tarsal solenidion (*ω*) 6, knobbed. **Leg II** 32, femur 10, basiventral femoral seta (*bv*) 14; genu 6, antaxial genual seta (*l"*) 11; tibia 8; tarsus 8, seta *ft’* 5, seta *ft"* 18, seta *u’* 6; tarsal empodium (*em*) 6, simple, 4-rayed, tarsal solenidion (*ω*) 6, knobbed. **Opisthosoma** dorsally with 31 annuli, with dark shading on rear annular margins, ventrally with 67 annuli, with round microtubercles. Setae *c2* 42 on ventral annulus 13, 58 apart; setae *d* 70 on ventral annulus 27, 40 apart; setae *e* 25 on ventral annulus 47, 20 apart; setae *f* 40 on ventral annulus 63, 30 apart. Setae *h1* 5, *h2* 130. **Male genitalia** 15, 25 wide, setae *3a* 18, 19 apart.

**Figure 24. F24:**
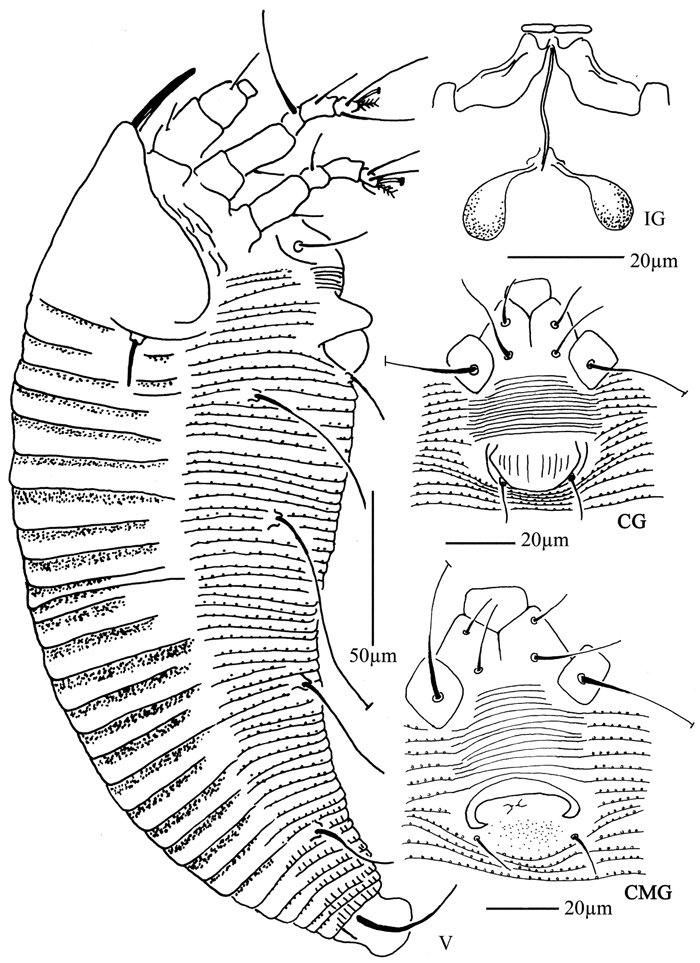
*Tetra simonia* sp. n.: **L** lateral view of female **IG** female internal genitalia **CG** coxae and female genitalia **CMG** coxae and male genitalia.

**Figure 25. F25:**
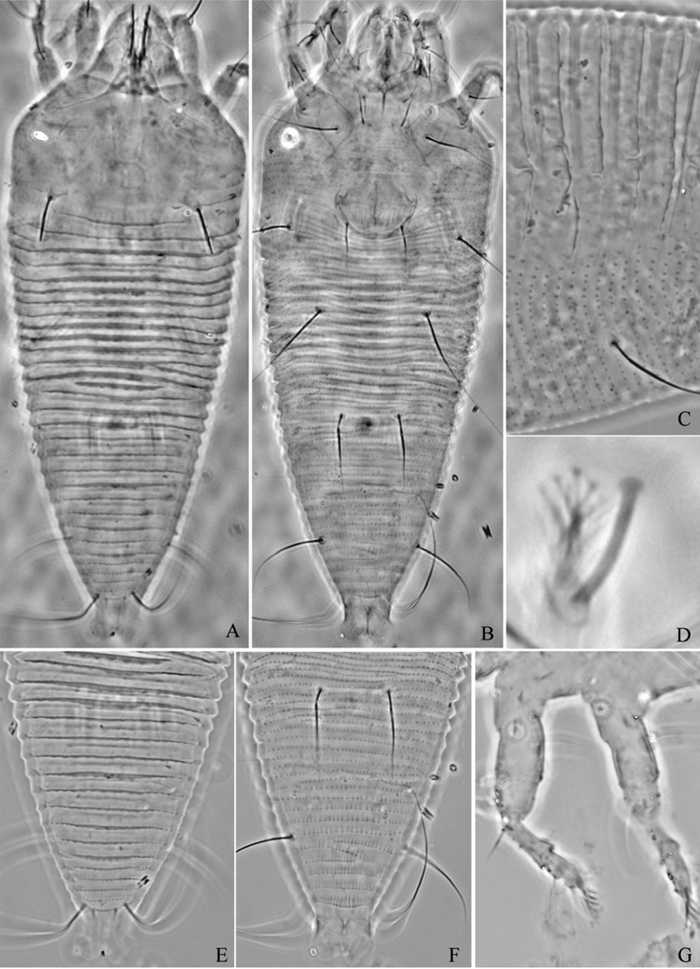
*Tetra simonia* sp. n.: **A** dorsal view of female **B** ventral view of female **C** lateral microtubercles **D **empodium **E** dorsal view of female posterior part **F** ventral view of female posterior part **G** leg I and leg II.

**Figure 26.**
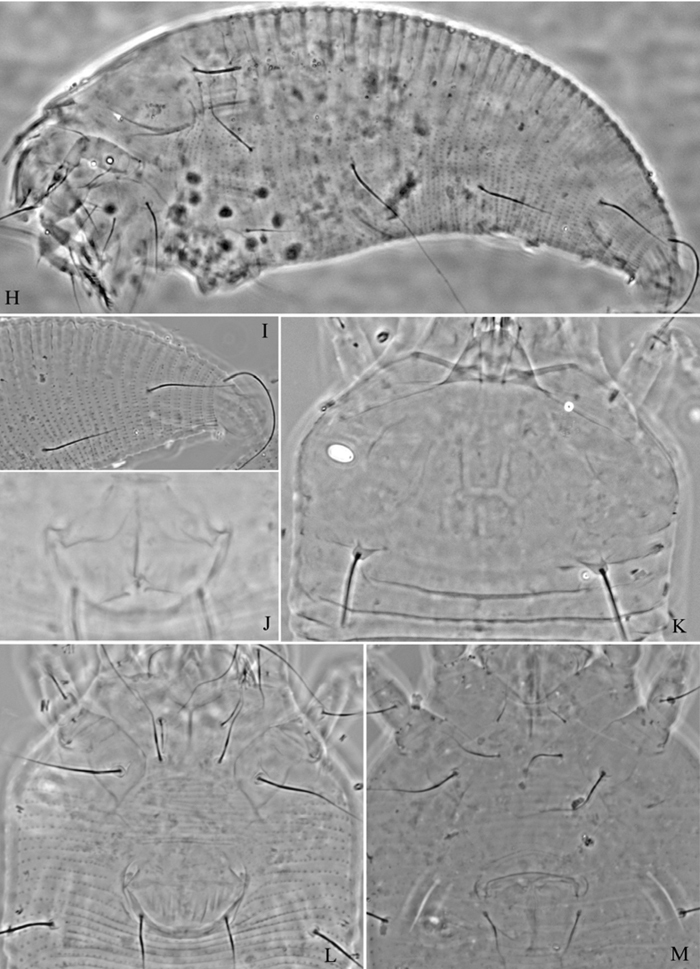
*Tetra simonia* sp. n.: **H** lateral view of female **I** lateral view of female posterior part **J** female internal genitalia **K** prodorsal shield **L** coxae and female genitalia **M** coxae and male genitalia.

##### Type material.

**Holotype**, female (slide number NJAUEri799, marked Holotype), from *Populus simonii* Carr. (Salicaceae), Xining, Qinghai Province, P. R. China, 36°38'18"N, 101°45'27"E, elevation 2241m, 21 July 2007, coll. Xiao-Feng Xue. **Paratypes**, 13 females and 1 male (slide number NJAUEri799), with the same data as holotype.

##### Additional material.

4 females (slide number NJAUEri789A), from *Populus simonii* Carr. (Salicaceae), Xining, Qinghai Province, P. R. China, 36°38'18"N, 101°45'27"E, elevation 2241m, 21 July 2007, coll. Xiao-Feng Xue. 4 females and 2 males (slide number NJAUEri823), from *Populus simonii* Carr. (Salicaceae), Beishan National Forest Park, Huzhu County, Qinghai Province, P. R. China, 36°53'35"N, 102°25'56"E, elevation 2610m, 22 July 2007, coll. Xiao-Feng Xue.

##### Relation to host.

Vagrant on leaf lower surface. No damage to the host was observed.

##### Etymology.

The specific designation *simonia* is from the species name of host plant, *simonii*; feminine in gender.

##### Differential diagnosis.

This species is similar to *Tetra smilaxis* Xue, Song & Hong, 2006a, but can be differentiated from the latter by opisthosoma with dark shading on rear annular margins (dark shading absent in *Tetra smilaxis*), prodorsal shield as wide as opisthosoma (opisthosoma wider than prodorsal shield in *Tetra smilaxis*), admedian lines connected at basal 1/3 but separate at basal 2/3 of prodorsal shield (admedian lines connected at basal 1/3 and basal 2/3 of prodorsal shield in *Tetra smilaxis*).

### Family Diptilomiopidae Keifer, 1944. Subfamily Diptilomiopinae Keifer, 1944. Genus *Diptacus* Keifer, 1951

#### Diptacus berberinus sp. n.

urn:lsid:zoobank.org:act:3EF374D2-74CD-46A0-833C-A80FC1EAE1E2

http://species-id.net/wiki/Diptacus_berberinus

**Figure 27.**
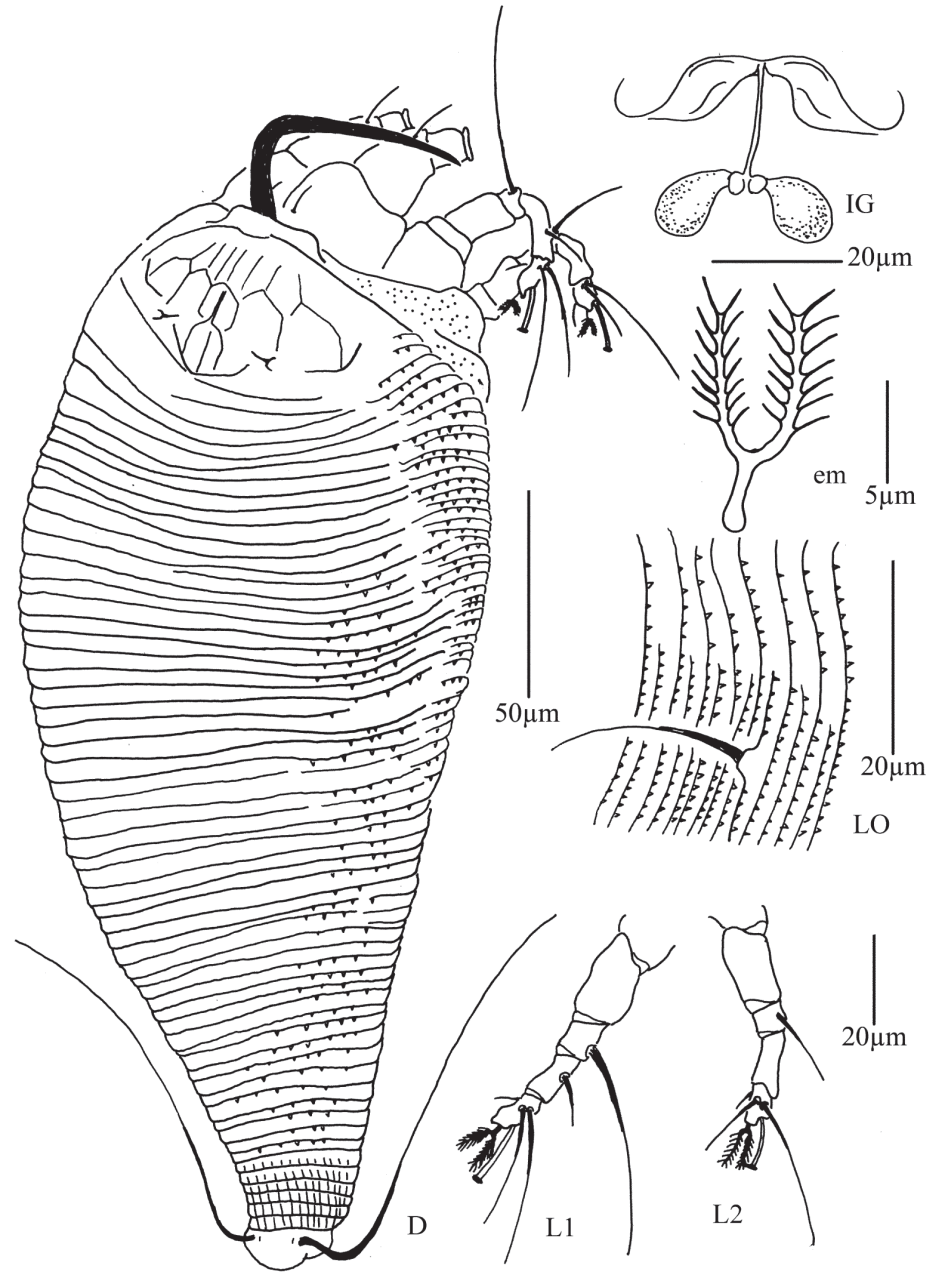
*Diptacus berberinus* sp. n.: **D** dorsal view of female **IG** female internal genitalia **LO** lateral microtubercles **L1** leg I **L2** leg II **em** empodium.

Figures 27 [Fig F28] [Fig F29] –30

##### Description.

Female. (n = 9) Body fusiform, light yellow, 283 (280–360), 110 (102–110) wide, 115 (114–115) thick. **Gnathosoma** 26 (25–27), projecting downwards, pedipalp coxal seta (*ep*) 6 (5–6), dorsal pedipalp genual seta (*d*) 12 (11–12), cheliceral stylets 65 (65–66). **Prodorsal shield** 40 (40–46), 80 (77–80) wide, with wide and broad frontal lobe, 7 (7–8); median, admedian and submedian lines present, admedian lines connected at the basal 1/3 and 2/3 of prodorsal shield, forming 3 cells on each side, submedian lines connected with the median and admedian at the basal 2/3 of prodorsal shield, forming the cell-like pattern at anterior shield margin. Scapular tubercles ahead of rear shield margin, 3 (2–3), 30 (27–30) apart, scapular setae (*sc*) 5 (4–5), projecting centrad to forward. **Coxigenital region** with 13 (13–15) annuli, with triangular microtubercles. Coxisternal plate I with granules, coxisternal plate II smooth, anterolateral setae on coxisternum **I** (*1b*) 20 (18–20), 17 (17–18) apart, proximal setae on coxisternum **I** (*1a*) 43 (43–45), 19 (17–19) apart, proximal setae on coxisternum **II** (*2a*) 70 (70–80), 49 (43–56) apart, tubercles *1b* and *1a* 12 (12–13) apart, tubercles *1a* and *2a* 17 (14–17) apart. Prosternal apodeme separated, 5 (5–6). **Leg I** 65 (60–65), femur 20 (20–22), basiventral femoral seta (*bv*) absent; genu 8 (8–9), antaxial genual seta (*l"*) 48 (48–52); tibia 18 (17–19), paraxial tibial seta (*l’*) 11 (10–11), located at 1/2 from dorsal base; tarsus 11 (11–12), seta *ft’* 30 (29–30), seta *ft"* 40 (37–40), seta *u’* 7 (6–7); tarsal empodium (*em*) 11 (10–11), divided, 7-rayed on each side, tarsal solenidion (*ω*) 10 (10–14), knobbed. **Leg II** 55 (54–55), femur 20 (19–20), basiventral femoral seta (*bv*) absent; genu 8 (7–8), antaxial genual seta (*l"*) 18 (15–18); tibia 17 (16–17); tarsus 11 (10–11), seta *ft’* 11 (10–11), seta *ft"* 44 (44–50), seta *u’* 8 (7–8); tarsal empodium (*em*) 11 (10–11), divided, 7-rayed on each side, tarsal solenidion (*ω*) 10 (10–11), knobbed. **Opisthosoma** dorsally with 59 (54–62) annuli, smooth, ventrally with 106 (101–106) annuli, with triangular microtubercles. Setae *c2* 115 (110–115) on ventral annulus 18 (18–20), 74 (74–75) apart; setae *d* 100 (100–120) on ventral annulus 40 (37–40), 56 (49–56) apart; setae *e* 60 (55–60) on ventral annulus 65 (60–65), 31 (29–35) apart; setae *f* 65 (60–70) on ventral annulus 93 (87–93), 35 (35–37) apart. Setae *h1* 2 (1–2), *h2* 103 (95–165). **Female genitalia** 35 (31–40), 35 (34–42) wide, coverflap with short lines on base, and 4 longitudinal ridges in 2 ranks, 1 ridge near the base and 3 ridges at distal margin, setae *3a* 12 (11–15), 24 (24–25) apart.

Male. (n = 1) Body fusiform, light yellow, 269, 86 wide. **Gnathosoma** 60, projecting downwards, pedipalp coxal seta (*ep*) 5, dorsal pedipalp genual seta (*d*) 11, cheliceral stylets 65. **Prodorsal shield** has the same design as female, 38, 71 wide, with wide and broad frontal lobe, 7. Scapular tubercles ahead of rear shield margin, 3, 27 apart, scapular setae (*sc*) 4, projecting centrad to forward. **Coxigenital region** with 14 annuli, with triangular microtubercles. Coxisternal plate I with granules, coxisternal plate II smooth, anterolateral setae on coxisternum **I** (*1b*) 22, 16 apart, proximal setae on coxisternum **I** (*1a*) 36, 16 apart, proximal setae on coxisternum **II** (*2a*) 60, 41 apart, tubercles *1b* and *1a* 11 apart, tubercles *1a* and *2a* 12 apart. Prosternal apodeme separated, 6. **Leg I** 46, femur 17, basiventral femoral seta (*bv*) absent; genu 7, antaxial genual seta (*l"*) 46; tibia 13, paraxial tibial seta (*l’*) 10, located at 1/2 from dorsal base; tarsus 8, seta *ft’* 30, seta *ft"* 37, seta *u’* 6; tarsal empodium (*em*) 10, divided, 7-rayed on each side, tarsal solenidion (*ω*) 10, knobbed. **Leg II** 38, femur 17, basiventral femoral seta (*bv*) absent; genu 7, antaxial genual seta (*l"*) 15; tibia 13; tarsus 6, seta *ft’* 10, seta *ft"* 38, seta *u’* 7; tarsal empodium (*em*) 10, divided, 7-rayed on each side, tarsal solenidion (*ω*) 11, knobbed. **Opisthosoma** dorsally with 56 annuli, smooth, ventrally with 83 annuli, with triangular microtubercles. Setae *c2* 95 on ventral annulus 15, 63 apart; setae *d* 100 on ventral annulus 30, 49 apart; setae *e* 55 on ventral annulus 46, 27 apart; setae *f* 60 on ventral annulus 71, 25 apart. Setae *h1* 2, *h2* 120. **Male genitalia** 23, 30 wide, setae *3a* 10, 23 apart.

**Figure 28. F28:**
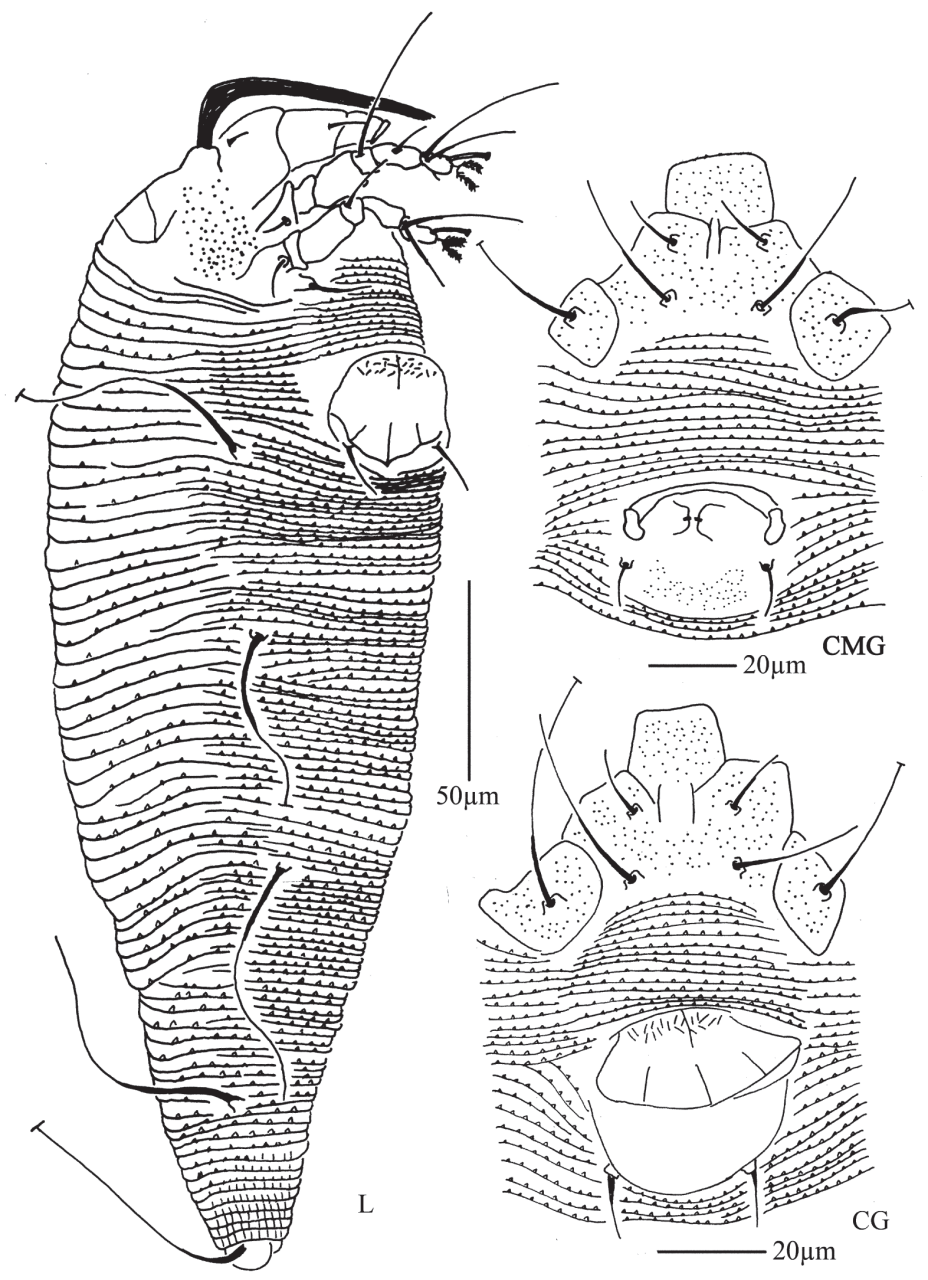
*Diptacus berberinus* sp. n.: **L** lateral view of female **CMG** coxae and male genitalia **CG** coxae and female genitalia.

**Figure 29. F29:**
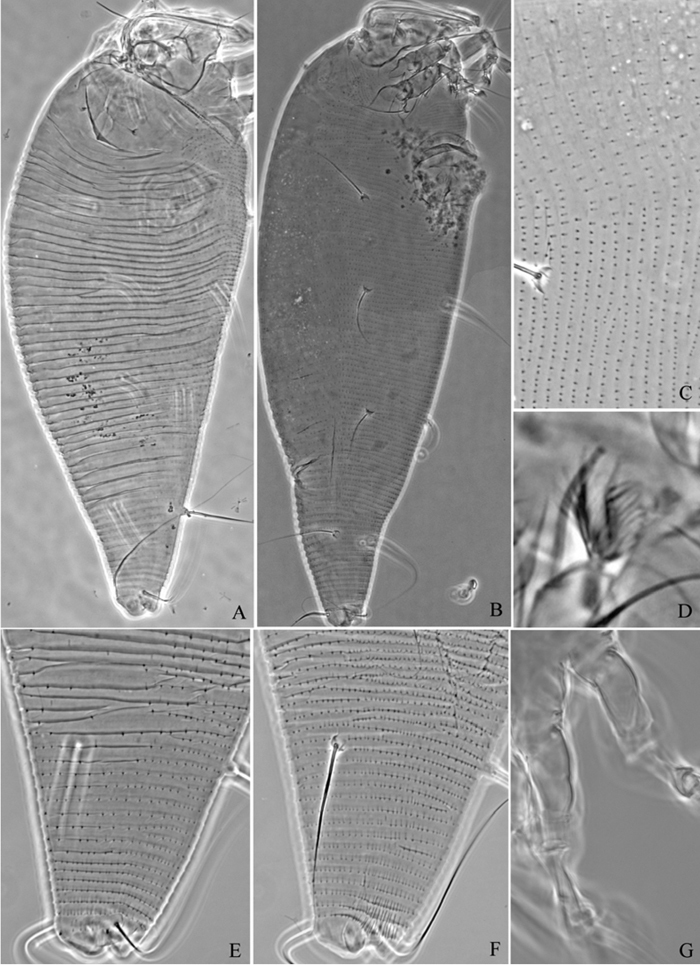
*Diptacus berberinus* sp. n.: **A** dorsal view of female **B** ventral view of female **C** lateral microtubercles **D** empodium **E** dorsal view of female posterior part **F** ventral view of female posterior part **G** leg I and leg II.

**Figure 30.**
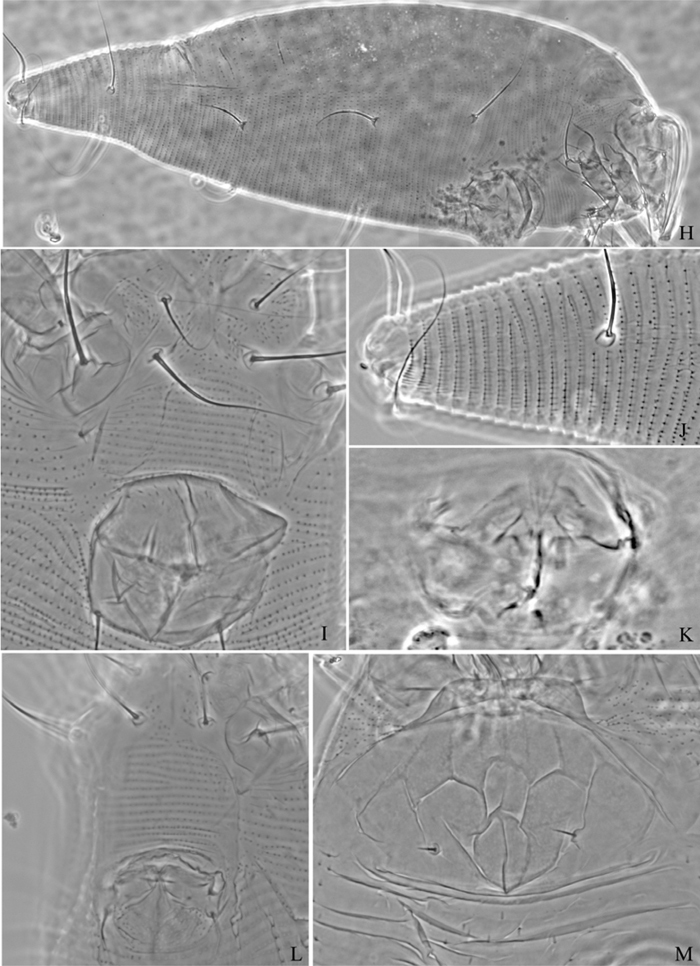
*Diptacus berberinus* sp. n.: **H** lateral view of female **I** coxae and female genitalia **J** lateral view of female posterior part **K** female internal genitalia **L** coxae and male genitalia **M** prodorsal shield.

##### Type material.

**Holotype**, female (slide number 783, marked Holotype), from *Berberis amurensis* Rupr.(Berberidaceae), Mengda Natural Reserve, Xunhua County, Qinghai Province, P. R. China, 35°47'38"N, 102°40'40"E, elevation 2523m, 19 July 2007, coll. Xiao-Feng Xue. **Paratypes**, 8 females and 1 male (slide number 783), with the same data as holotype.

##### Relation to host.

Vagrant on leaf lower surface. No damage to the host was observed.

##### Etymology.

The specific designation *berberinus* is from the generic name of host plant, *Berberis*; masculine in gender.

##### Differential diagnosis.

This species is similar to *Diptacus maddenis* Song, Xue & Hong, 2007a, but can be differentiated from the latter by opisthosomal dorsal annuli smooth (opisthosomal dorsal annuli with elongated microtubercles in *Diptacus maddenis*), female genital coverflap with short lines at the base (genital coverflap with granules in *Diptacus maddenis*), tarsal empodium 7-rayed (4-rayed in *Diptacus maddenis*).

#### Diptacus mengdaensis sp. n.

urn:lsid:zoobank.org:act:12904959-7EE9-4780-BCC1-EF886FA54F98

http://species-id.net/wiki/Diptacus_mengdaensis

**Figure 31.**
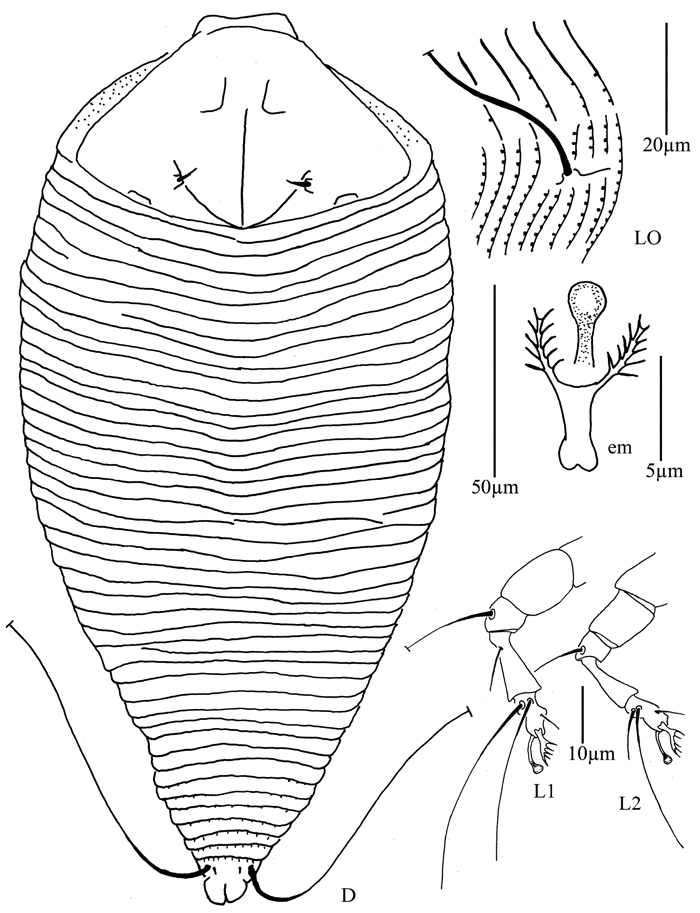
*Diptacus mengdaensis* sp. n.: **D** dorsal view of female **LO** lateral microtubercles **em** empodium **L1** leg I **L2** leg II.

Figures 31 [Fig F32] –33

##### Description.

Female. (n = 13) Body fusiform, light yellow, 215 (210–232), 104 (104–105) wide, 71 (71–74) thick. **Gnathosoma** 25 (24–25), projecting downwards, pedipalp coxal seta (*ep*) 4 (4–5), dorsal pedipalp genual seta (*d*) 14 (14–15), cheliceral stylets 62 (61–62). **Prodorsal shield** 50 (50–51), 76 (71–76) wide, with wide and broad frontal lobe, 7 (7–8); median, admedian and submedian lines present, admedian lines connected at the base of prodorsal shield, ending at basal 1/3 of prodorsal shield. Scapular tubercles ahead of rear shield margin, 3 (3–4), 34 (34–35) apart, scapular setae (*sc*) 6 (6–7), projecting centrad. **Coxigenital region** with 16 (15–16) annuli, with microtubercles. Coxisternal plates with short lines, anterolateral setae on coxisternum **I** (*1b*) 16 (16–18), 18 (18–20) apart, proximal setae on coxisternum **I** (*1a*) 25 (25–26), 17 (17–18) apart, proximal setae on coxisternum **II** (*2a*) 60 (60–70), 44 (44–45) apart, tubercles *1b* and *1a* 12 (12–13) apart, tubercles *1a* and *2a* 16 (16–17) apart. Prosternal apodeme separated, 10 (9–10). **Leg I** 60 (58–60), femur 16 (16–17), basiventral femoral seta (*bv*) absent; genu 10 (8–10), antaxial genual seta (*l"*) 52 (52–53); tibia 17 (16–17), paraxial tibial seta (*l’*) 10 (9–10), located at 1/3 from dorsal base; tarsus 11 (10–11), seta *ft’* 30 (25–30), seta *ft"* 40 (32–40), seta *u’* 6 (5–6); tarsal empodium (*em*) 8 (8–9), divided, 5-rayed at each side, tarsal solenidion (*ω*) 9 (9–10), knobbed. **Leg II** 54 (50–54), femur 18 (17–18), basiventral femoral seta (*bv*) absent; genu 7 (7–8), antaxial genual seta (*l"*) 16 (15–16); tibia 11 (9–11); tarsus 8 (8–10), seta *ft’* 12 (12–13), seta *ft"* 34 (34–34), seta *u’* 5 (5–6); tarsal empodium (*em*) 9 (8–9), divided, 5-rayed at each side, tarsal solenidion (*ω*) 9 (9–10), knobbed. **Opisthosoma** dorsally with 44 (44–48) annuli, smooth, ventrally with 112 (112–115) annuli, with round microtubercles. Setae *c2* 55 (54–55) on ventral annulus 16 (16–18), 76 (70–76) apart; setae *d* 90 (90–93) on ventral annulus 39 (39–42), 51 (51–53) apart; setae *e* 70 (65–70) on ventral annulus 67 (67–69), 29 (29–31) apart; setae *f* 50 (50–55) on ventral annulus 98 (98–101), 37 (36–37) apart. Setae *h1* 2 (2–3), *h2* 152 (150–152). **Female genitalia** 24 (24–25), 37 (37–39) wide, coverflap with 4 longitudinal ridges in 2 ranks, 1 near the base and 3 at distal margin, setae *3a* 12 (10–12), 20 (20–21) apart.

Male. Unknown.

**Figure 32. F32:**
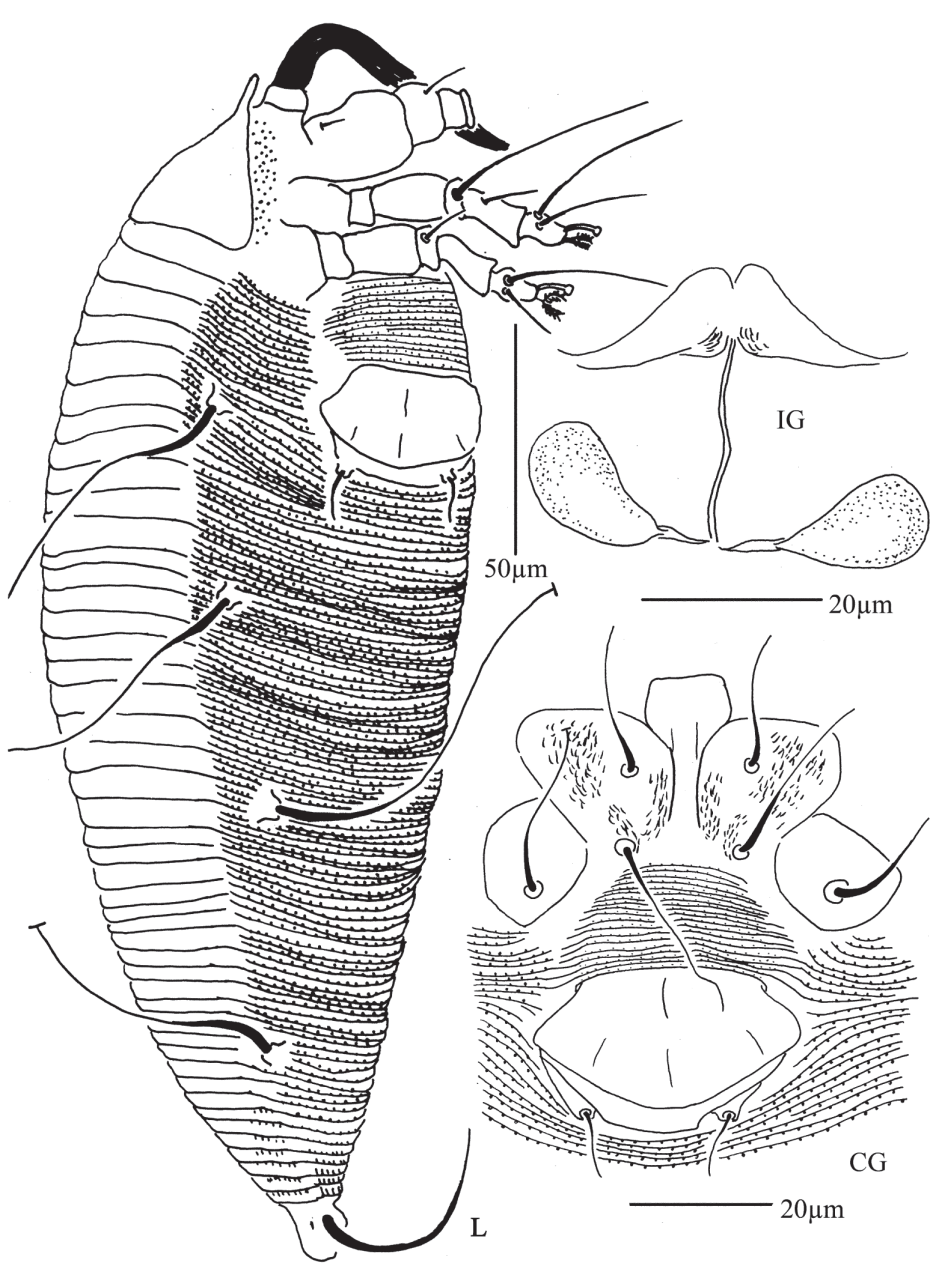
*Diptacus mengdaensis* sp. n.: **L** lateral view of female **IG** female internal genitalia **CG** coxae and female genitalia.

**Figure 33.**
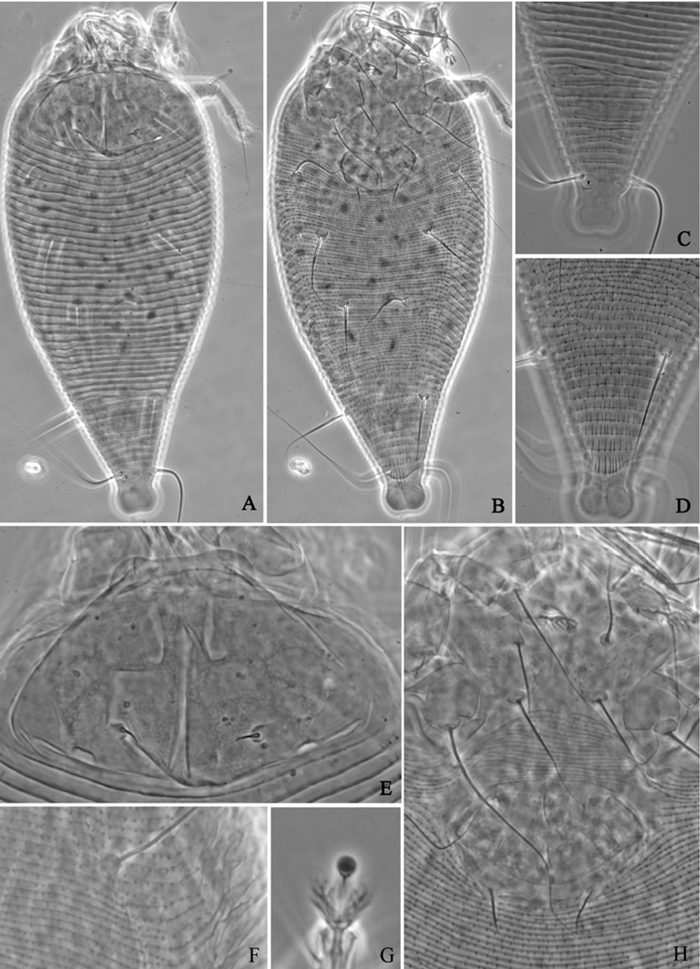
*Diptacus mengdaensis*sp. n.: **A** dorsal view of female **B** ventral view of female **C** dorsal view of female posterior part **D** ventral view of female posterior part **E** prodorsal shield **F** lateral microtubercles **G** empodium **H** coxae and female genitalia.

##### Type material.

**Holotype**, female (slide number NJAUEri777, marked Holotype), from *Lonicera elisae* Franch. (Caprifoliaceae), Mengda Natural Reserve, Xunhua County, Qinghai Province, P. R. China, 35°47'38"N, 102°40'40"E, elevation 2523m, 19 July 2007, coll. Xiao-Feng Xue. **Paratypes**, 12 females (slide number NJAUEri777), with the same data as holotype.

##### Relation to host.

Vagrant on leaf lower surface. No damage to the host was observed.

##### Etymology.

The specific designation *mengdaensis* is from the place name Mengda Natural Reserve, where this new species was collected; feminine in gender.

##### Differential diagnosis.

This species is similar to *Diptacus lonicerae* Kuang, 2001, but can be differentiated from the latter by prodorsal shield with admedian lines and submedian lines separated (admedian lines and submedian lines connected in *Diptacus lonicerae*), prodorsal shield frontal lobe wide and broad (frontal lobe small in *Diptacus lonicerae*), female genital coverflap with 4 longitudinal ridges in 2 ranks, 1 near the base and 3 far from the base (female genital coverflap with 6–8 longitudinal ridges in *Diptacus lonicerae*).

### Subfamily Rhyncaphytoptinae Roivainen, 1953. Genus *Rhyncaphytoptus* Keifer, 1939

#### Rhyncaphytoptus ulmi

Xin & Dong, 1981

http://species-id.net/wiki/Rhyncaphytoptus_ulmi

Rhyncaphytoptus ulmi Xin and Dong 1981: 216–217, figures 2–3.
Rhyncaphytoptus ulmi ; Amrine and Stasny 1994: 277.
Rhyncaphytoptus ulmi ; Amrine and Stasny 1996: 300.
Rhyncaphytoptus ulmi ; Hong and Zhang 1996: 79, figures 188–1–188–2.
Rhyncaphytoptus ulmi ; Kuang et al. 2005: 155–156, figure 158.
Rhyncaphytoptus ulmi ; Xue et al. 2006b: 3.
Rhyncaphytoptus ulmi ; Song et al. 2007b: 59.
Rhyncaphytoptus ulmi ; Xue et al. 2009: 3.

##### Material examined.

16 females (slide number NJAUEri792B and NJAUEri795), from *Ulmus* sp. (Ulmaceae), Xining, Qinghai Province, P. R. China, 36°38'18"N, 101°45'27"E, elevation 2241m, 21 July 2007, coll. Xiao-Feng Xue.

##### Host.

*Ulmus* sp. (Ulmaceae).

##### Relation to host.

Vagrant on leaf lower surface. No damage to the host was observed.

##### Distribution.

China (Jiangsu, Gansu, Jilin, Liaoning, Shaanxi, Shandong, Xinjiang, Qinghai).

#### Rhyncaphytoptus spinus sp. n.

urn:lsid:zoobank.org:act:F3227702-A360-40D4-9367-E395D5F361DE

http://species-id.net/wiki/Rhyncaphytoptus_spinus

**Figure 34.**
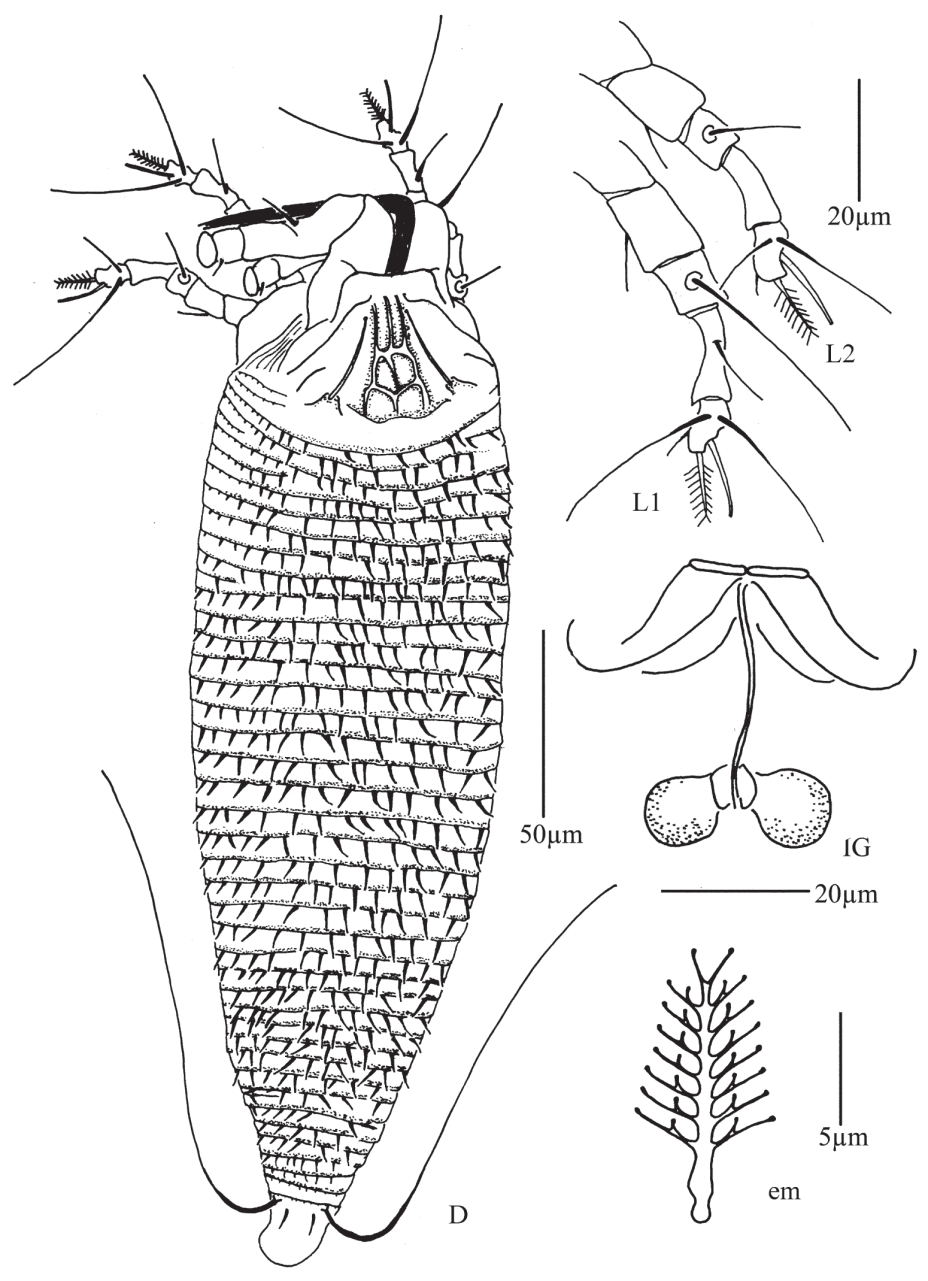
*Rhyncaphytoptus spinus* sp. n.: **D** dorsal view of female **L1** leg I **L2** leg II **IG** female internal genitalia **em** empodium.

Figures 34 [Fig F35] –36

##### Description.

Female. (n = 6) Body fusiform, light yellow, 270 (232–323), 82 (82–91) wide, 105 (104–105) thick. **Gnathosoma** 58 (56–59), projecting downwards, suboral plate present, pedipalp coxal seta (*ep*) 4 (4–5), dorsal pedipalp genual seta (*d*) 10 (9–11), palp tarsus ventral seta (*v*) 4 (3–4), cheliceral stylets 71 (71–72). **Prodorsal shield** 38 (38–40), 60 (57–64) wide, with broad frontal lobe, 7 (6–7); median, admedian and submedian lines present, median, admedian lines connected at basal 1/3 and 2/3 of prodorsal shield, forming three cells each both side. Scapular tubercles ahead of rear shield margin, 6 (5–6), 34 (34–40) apart, scapular setae (*sc*) 21 (21–25), projecting forward, knobbed at the end. **Coxigenital region** with 15 (14–15) annuli, with microtubercles. Coxisternal plates smooth, anterolateral setae on coxisternum **I** (*1b*) 28 (26–28), 16 (16–18) apart, proximal setae on coxisternum **I** (*1a*) 46 (44–46), 10 (10–13) apart, proximal setae on coxisternum **II** (*2a*) 80 (71–80), 30 (30–36) apart, tubercles *1b* and *1a* 6 (5–7) apart, tubercles *1a* and *2a* 9 (9–13) apart. Prosternal apodeme combined, 6 (6–7). **Leg I** 44 (43–49), femur 16 (16–17), basiventral femoral seta (*bv*) 18 (15–20); genu 8 (8–9), antaxial genual seta (*l"*) 30 (30–32); tibia 12 (12–13), paraxial tibial seta (*l’*) 14 (14–17), located at 1/3 from dorsal base; tarsus 9 (9–10), seta *ft’* 26 (24–28), seta *ft"* 35 (34–35), seta *u’* 8 (6–8); tarsal empodium (*em*) 12 (12–13), simple, 8-rayed, tarsal solenidion (*ω*) 11 (10–11), tapered. **Leg II** 41 (41–46), femur 14 (14–16), basiventral femoral seta (*bv*) 18 (18–19); genu 7 (7–8), antaxial genual seta (*l"*) 10 (10–11); tibia 11 (10–11); tarsus 8 (8–10), seta *ft’* 12 (12–13), seta *ft"* 34 (34–34), seta *u’* 7 (6–7); tarsal empodium (*em*) 12 (12–13), simple, 8-rayed, tarsal solenidion (*ω*) 11 (10–11), tapered. **Opisthosoma** dorsally with 38 (38–42) annuli, with long spiny microtubercles, ventrally with 98 (97–98) annuli, with triangle microtubercles. Setae *c2* 33 (31–33) on ventral annulus 17 (17–19), 76 (71–76) apart; setae *d* 97 (95–97) on ventral annulus 38 (38–40), 53 (53–69) apart; setae *e* 41 (41–45) on ventral annulus 60 (59–61), 31 (31–39) apart; setae *f* 36 (36–38) on ventral annulus 92 (92–93), 28 (28–30) apart. Setae *h1* 5 (5–6), *h2* 110 (110–115). **Female genitalia** 21 (18–21), 32 (32–35) wide, coverflap smooth, setae *3a* 75 (70–75), 20 (20–23) apart.

Male. Unknown.

**Figure 35. F35:**
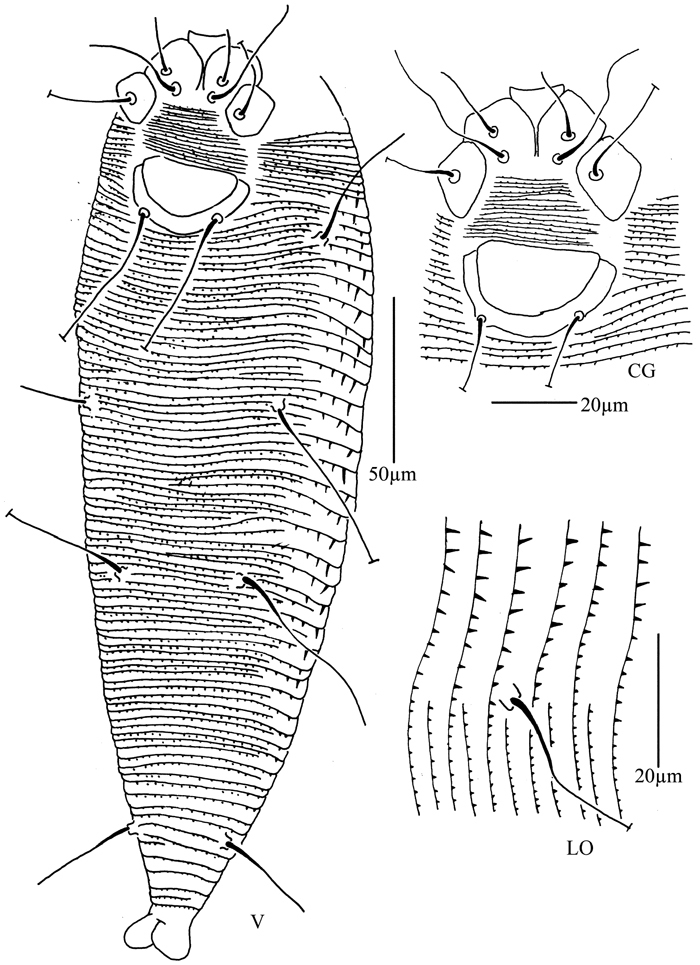
*Rhyncaphytoptus spinus* sp. n.: **V** ventral view of female **CG** coxae and female genitalia **LO** lateral microtubercles.

**Figure 36.**
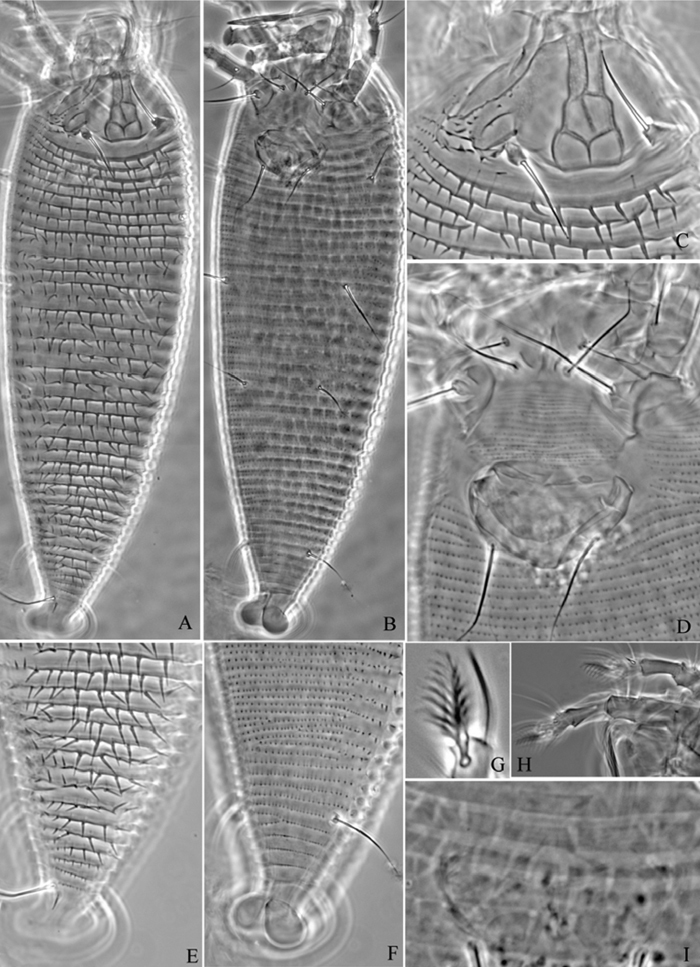
*Rhyncaphytoptus spinus* sp. n.: **A** dorsal view of female **B** ventral view of female **C** prodorsal shield **D** coxae and female genitalia **E** dorsal view of female posterior part **F** ventral view of female posterior part **G** empodium **H** leg I and leg II **I** female internal genitalia.

##### Type material.

**Holotype**, female (slide number NJAUEri820, marked Holotype), from *Lonicera rupicola* Hook. f. et Thoms. (Caprifoliaceae), Beishan National Forest Park, Huzhu County, Qinghai Province, P. R. China, 36°53'35"N, 102°25'56"E, elevation 2610m, 22 July 2007, coll. Xiao-Feng Xue. **Paratypes**, 5 females (slide number NJAUEri820), with the same data as holotype.

##### Relation to host.

Vagrant on leaf lower surface. No damage to the host was observed.

##### Etymology.

The specific designation *spinus* is from the character of the dorsal opisthosomal microtubercles, spiny, “spina, spinus” in Latin; masculine in gender.

##### Differential diagnosis.

This species is similar to *Rhyncaphytoptus guanegounis* Song, Xue & Hong, 2007b, but can be differentiated from the latter by median, admedian and submedian lines present on prodorsal shield (prodorsal shield smooth in *Rhyncaphytoptus guanegounis*), prodorsal shield with wide and broad frontal lobe (prodorsal shield with long and broad frontal lobe in *Rhyncaphytoptus guanegounis*), opisthosomal dorsal annuli with spiny microtubercles (opisthosomal dorsal annuli smooth in *Rhyncaphytoptus guanegounis*), tarsal empodium (*em*) 8-rayed (6-rayed in *Rhyncaphytoptus guanegounis*).

## Discussion

Although Qinghai has climate, vegetation and biological diversity similar to Tibet, we did not find any eriophyoid mite species already reported in Tibet (Song et al. 2011). On the contrary, 6 of 23 species were reported from Gansu Province or Shaanxi Province, east neighboring provinces. As of 2010, 932 eriophyoid mite species have been described from China (Hong et al. 2010), and the number is still increasing. Qinghai has about 8% of the land of China, but only about 2% eriophyoid mites were reported to date. Furthermore, only two investigations have been conducted. More systematic collections of eriophyoid mites from Qinghai Province are needed in the near future.

## Supplementary Material

XML Treatment for Aceria paramacrodonis

XML Treatment for Aceria qinghaiensis

XML Treatment for Acaphyllisa tuberculumae

XML Treatment for Proiectus xiningensis

XML Treatment for Phyllocoptes beishaniensis

XML Treatment for Phyllocoptes asperatae

XML Treatment for Phyllocoptes dangchangi

XML Treatment for Phyllocoptes gansuensis

XML Treatment for Phyllocoptruta platyclada

XML Treatment for Aculus changbais

XML Treatment for Aculus huangzhongensis

XML Treatment for Aculodes salicis

XML Treatment for Aculops ulmi

XML Treatment for Aculops xiningensis

XML Treatment for Tetraspinus syringae

XML Treatment for Tetra pinnatifidae

XML Treatment for Tetra pruniana

XML Treatment for Tetra pyriana

XML Treatment for Tetra simonia

XML Treatment for Diptacus berberinus

XML Treatment for Diptacus mengdaensis

XML Treatment for Rhyncaphytoptus ulmi

XML Treatment for Rhyncaphytoptus spinus
